# CCR8 antagonist suppresses liver cancer progression via turning tumor-infiltrating Tregs into less immunosuppressive phenotype

**DOI:** 10.1186/s13046-025-03286-x

**Published:** 2025-04-04

**Authors:** Binle Tian, Zhilong Wang, Mei Cao, Na Wang, Xuebing Jia, Yuanyuan Zhang, Jingyi Zhou, Sijia Liu, Wen Zhang, Xiao Dong, Zheng Li, Junli Xue, JianFei Wang, Guo-Huang Fan, Qi Li

**Affiliations:** 1 Cancer Center, Shanghai General Hospital, Shanghai Jiao Tong University School of Medicine, Shanghai, 200080 China; 2 Shanghai Key Laboratory of Pancreatic Disease, Institute of Pancreatic Disease, Shanghai Jiao Tong University School of Medicine, Shanghai, 200080 China; 3 Department of Oncology, Immunophage Biotech Co., Ltd., 10 Lv Zhouhuang Road, Shanghai, 201114 China; 4 Department of Gynecology and Obstetrics, Taihe Hospital, Hubei University of Medicine, Shiyan, 442000 China; 5 Department of Antibody Development, Immunophage Biotech Co., Ltd., 10 Lv Zhouhuang Road, Shanghai, 201114 China; 6 Department of Medical Oncology, Shanghai Pulmonary Hospital, Tongji University School of Medicine, Shanghai, 200433 China; 7 Department of Autoimmune Disease, Immunophage Biotech Co., Ltd., 10 Lv Zhouhuang Road, Shanghai, 201114 China; 8 Department of Oncology, Shanghai East Hospital, Tongji University School of Medicine, Shanghai, 200123 China; 9 Excecutive Office, Immunophage Biotech Co., Ltd., 10 Lv Zhouhuang Road, Shanghai, 201114 China; 10 Shanghai Laboratory Animal Research Center, Shanghai, 201203 China

**Keywords:** CCR8, IPG0521m, Tregs, Liver cancer, Single-cell sequencing, Tumor microenvironment

## Abstract

**Background:**

Regulatory T cells (Tregs) are the main immunosuppressive cells in tumor immune microenvironment (TIME). However, systemic Treg depletion is not favored due to the crucial role of Tregs in the maintenance of immune homeostasis and prevention of autoimmunity. Recently, CCR8 has been identified as a key chemokine receptor expressed on tumor-infiltrating Tregs and targeted blockade of CCR8 exerts anticancer effect in several cancer types, but whether this pathway is involved in the progression of hepatocellular carcinoma (HCC) remains unclear.

**Methods:**

We determined the involvement of CCR8^+^ Tregs in HCC using human HCC tissues and TCGA database, and examined the anticancer effect and the underlying molecular mechanisms of the CCR8 antagonist, IPG0521m, which was developed in house, in murine liver cancer model with flow cytometry, bulk and single-cell RNA sequencing and Real-Time PCR.

**Results:**

Remarkable increase in CCR8^+^ Tregs was observed in human HCC tissues. Treatment of syngeneic liver cancer model with IPG0521m resulted in dramatic inhibition of tumor growth, associated with increased CD8^+^ T cells in tumor tissues. Bulk RNA sequencing analysis indicated that IPG0521m treatment resulted in remarkable increase in antitumor immunity. Furthermore, single-cell RNA sequencing analysis demonstrated that IPG0521m treatment resulted in a switch of Tregs from high immunosuppression to low immunosuppression phenotype, associated with elevated CD8^+^ T and NK cell proliferation and cytotoxicity, and decreased myeloid-derived suppressor cells and tumor-associated macrophages in the tumor tissues.

**Conclusions:**

IPG0521m inhibited liver cancer growth via reducing the immunosuppressive function of Tregs, thereby boosting anti-cancer immunity. Our study paves the way for the clinical study of CCR8 antagonist in HCC and other cancers.

**Graphical Abstract:**

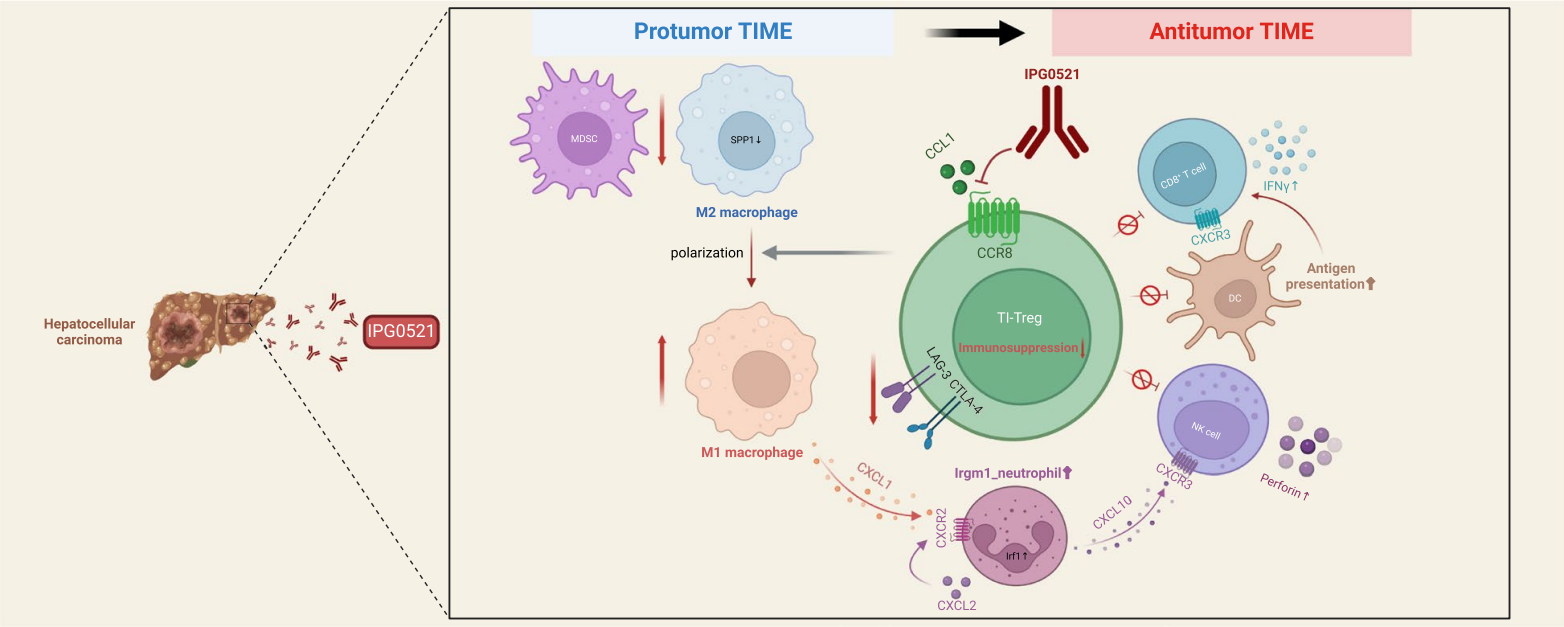

**Supplementary Information:**

The online version contains supplementary material available at 10.1186/s13046-025-03286-x.

## Introduction

Liver cancer is the sixth most common cancer and the fourth leading cause of cancer-related death worldwide [1]. Over 90% of liver cancer is hepatocellular carcinoma (HCC). For a long time, Sorafenib monotherapy has been the first-line treatment for advanced HCC, but with numerous side effects. In recent years, the emergence of immune checkpoint inhibitors has revolutionized HCC therapy. Antibodies targeting programmed cell death protein 1 (PD-1) or programmed cell death ligand 1 (PD-L1) have achieved unprecedented success in HCC. Moreover, the combination of anti-PD-L1 and anti-vascular endothelial growth factor has significantly improved the overall survival of HCC patients. However, great challenges remain due to the low remission rate of the current immunotherapy. Given the factor that the PD-1/PD-L1 axis is not enough to stimulate an effective antitumor immunity due to the immunosuppressive nature of the tumor microenvironment (TME), a better understanding of the tumor immune microenvironment (TIME) is urgently needed to discover new targets for more effective cancer immunotherapy.

In TIME, one of the key players that suppress anticancer immune response is regulatory T cell (Treg), which is characterized by the expression of forkhead box protein p3 (Foxp3). Tregs participate in the development and progression of tumors by inhibiting antitumor immunity through multiple mechanisms, including cytokine secretion, immune checkpoint molecules, and metabolic modulation [2]. In terms of HCC, tumor-resident Tregs express several immune checkpoints, including TIGIT and CTLA4 [3], and the increased percentage of Tregs is closely related to the tumor stage and tumor size of HCC [4]. These findings lead to the hypothesis that Tregs play a role in promoting the invasion and progression of HCC. However, given the essential role of Tregs in the maintenance of immune homeostasis, systemic Treg depletion may cause autoimmune disease. Hence, targeted inhibition or depletion of tumor-associated Tregs without globally compromising self-tolerance is a potential therapeutic approach for HCC.

Recent studies have shown that the chemokine receptor CCR8 is overexpressed in tumor-infiltrating Tregs of patients with different cancers, including breast, colon, and lung cancers, with no major CCR8-positivity found on peripheral Tregs [5–7]. These findings suggest that targeted inhibition or depletion of CCR8^+^ Tregs is a promising therapeutic strategy for cancer. However, intense disputes occur regarding the role of CCR8 in Treg's functions. A few studies using CCR8 knockout mice indicated that CCR8 is dispensable for their accumulation and immunosuppression [8]. Following this clue, several studies demonstrated that targeted depletion of CCR8^+^ Tregs with enhanced antibody-dependent cell-mediated cytotoxicity (ADCC) strategy induced potent anticancer response and synergized with anti-PD-1 treatment in several murine cancer models [9–13]. However, as CCR8 is expressed not only on Tregs, but also on Th2 cells, Th17 cells, central memory CD4^+^ and CD8^+^ T cells, NKT cells, monocytes, vascular endothelial cells, and tissues such as bladder and thymus [14–17], enhanced ADCC would inevitably cause severe side effects. In striking contrast, CCR8 has been shown to be critical for Treg immunosuppression. Stimulation of CCR8^+^ Tregs with CCL1 induces STAT3-dependent up-regulation of FOXp3, CD39, IL-10, and granzyme B, resulting in enhanced suppressive activity of these cells [18]. In gastric cancer, tumor-infiltrated Tregs with higher expression of CCR8 produce more IL-10 molecules in vitro. CCR8 blockade downregulates Treg-produced IL-10, and reverses the suppressive function of Tregs on the secretion and proliferation of CD8^+^ T cells [19]. In the muscle-invasive bladder cancer model, CCR8 blockade destabilizes intratumoral Tregs into a fragile phenotype, associated with reactivation of antitumor immunity and augment of anti-PD-1 therapeutic benefit [20]. Given these findings, CCR8 antagonism may offer therapeutic benefits to cancer patients without causing such side effects as that induced by the ADCC strategy. However, whether CCR8^+^ Tregs are involved in HCC, and if yes, whether blockade of CCR8 suppresses HCC progression remains unclear.

In the present study, we attempted to determine the potential involvement of CCR8^+^ Tregs in human HCC tissues and to examine the anticancer effect and the underlying molecular mechanisms of the CCR8 antagonistic antibody, IPG0521m, in murine liver cancer model. Our results demonstrated a marked increase in CCR8^+^ Tregs in human HCC tissues. Treatment of syngeneic liver cancer model with IPG0521m resulted in dramatic inhibition of tumor growth. Though the proportion of tumor-infiltrated Tregs remained unchanged, a marked increase in CD8^+^ T infiltration was observed in response to IPG0521m treatment. Single-cell RNA sequencing analysis revealed that IPG0521m treatment resulted in conversion of the tumor-infiltrating Tregs into a less immunosuppressive phenotype, associated with elevated anticancer immunity. Our study paves the way for the clinical study of CCR8 antagonists in HCC and other cancers.

## Materials and methods

### Animal studies

All procedures involving the care and use of animals in the study were reviewed and approved by the Institutional Animal Care and Use Committee of Immunophage Biotech and Shanghai Jiao Tong University School of Medicine. During the study, the care and use of animals were conducted in accordance with the guidance of the Association for Assessment and Accreditation of Laboratory Animal Care International (AAALAC). SJL mice were purchased from Beijing Vital River, Inc. (Beijing, China), and BALB/c mice were purchased from Jihui Biotech Ltd (Shanghai, China). The indicated stable cells (1 × 10^6^) were subcutaneously injected into the right flank abdomen of BALB/c mice in each group. Tumor size was measured twice a week, and mice were sacrificed to analyze the tumor burden after 2–4 weeks, and the tumor volume was calculated with the following formula: V = (length × width^2^)/2.

In some experiments, CD8^+^ T cell depletion was achieved through intraperitoneal administration of anti-mouse CD8α (BE0061, BioXCell) at 200 µg/mouse every 3 days.

### Human samples

The tumor sample collection protocol was approved by the Health Research Ethics Board of Shanghai General Hospital (No. 2022SQ164). Cancer patients with 18–80 years of age, who were scheduled for tumor resection surgery, were informed and signed the informed consent before their resected tumor tissues were collected. The resected tumor or paratumor tissues were stored in a tissue preservation solution.

### Immunohistochemistry

Human HCC tumor tissues and paired paratumor tissues chip (HLivH180Su16, Shanghai Outdo Biotech Company) were used, and patients’ information was collected. For IHC staining, deparaffinized and rehydrated tissue sections were blocked and incubated with a primary antibody against CCR8 (KB0001, Cobiotech, China), followed by incubation with a secondary antibody. DAB color was developed with diaminobenzene and hematoxylin. Slides were pictured with microscopy and viewed with a K-viewer. All slides were examined randomly by two independent pathologists, and IHC outcomes were determined by stained-positive cell counts. CCR8 positive cell counts were enumerated as the mean value of 5 randomized windows of 0.02 mm^2^ in high power magnification fields (HPF, 200 × magnification) of each section. CCR8^+^ cell density was counted as cells/mm^2^. For the density of CCR8 positive cells, the values below 40 cells/mm^2^ were defined as low ones, and the values above 200 cells/mm^2^ were defined as high ones. The clinical features of these samples with values ranging between 40 and 200 cells/mm^2^ were chaotic.

### Cell lines and cell culture

H22 cells, obtained from the Shanghai Chinese Academy of Sciences cell bank (China), were cultured in RPMI-1640 medium (Life Technologies, Carlsbad, CA, USA), supplemented with penicillin G (100 U/mL), streptomycin (100 mg/mL), and 10% fetal bovine serum (FBS, Life Technologies), at 37 °C in a humidified atmosphere with 5% CO_2_. 293 T and CHOK1 cells were obtained from EK-Bioscience. 293 T cells were cultured in Dulbecco’s Modified Eagle Medium (DMEM) with 1% sodium pyruvate containing 10% FBS, whereas CHOK1 cells were cultured in F12K Medium containing 10% FBS. The methods for the generation of gene-transfected cells are described in the Supplementary Materials. All cell lines were used within 10 passages after the first thawing.

### Screening and characterization of CCR8 antagonistic antibodies

Human CCR8 (hCCR8)−293 T cells or hCCR8-CHOK1 (2 × 10^7^/ml) were used to immunize 6–8 weeks-old SJL mice via abdominal and pedicle injection every two weeks. Splenocytes and mouse myeloma cells (Sp2/0-Ag14, Immunophage Institution) were fused using the polyethylene glycol (PEG) method, and selection was conducted in a medium containing hypoxanthine, aminopterin, and thymidine. Antibody clones were primarily screened using CCR8 binding and CCL1-CCR8 neutralizing assays. Supernatants containing antibodies were subjected to cell-based ELISA for reactivity with CCR8 overexpressing cells. Supernatants of these positive clones were then confirmed by fluorescence-activated cell sorting (FACS). To verify the specificity of the antibodies, the same supernatants were subjected to the cell-based ELISA with non-transfected parental cells. To screen the CCR8 antagonistic antibodies, antibodies with high binding affinity were subjected to Ca^2+^ mobilization assay using human CCR8 expressing 293 T cells stably transfected with genes of a Ca^2+^ indicator. After incubation of the CCR8–expressing cells with 90 nmol/L human CCL1 (R&D), Ca^2+^ influx was measured using a Fluorometric Imaging Plate Reader (FLIPR, Molecular Devices). The CCR8 antagonistic antibodies were sequenced, and their complementarity-determining regions were grafted onto the human framework. The potent and selective CCR8 antagonistic antibody identified in these processes was named IPG0521, which blocks CCR8 signaling with a single digital nanomolar IC_50_.

### Isolation of tumor-infiltrating Tregs

The tumor-bearing mice with tumor sizes ranging from 500 to 1000 mm^3^ were euthanized, and tumor tissues were harvested. The tumor tissues were minced into 1–2 mm^3^ pieces using dissecting laboratory scissors before being enzymatically digested (Mouse Tumor Dissociation Kit, Miltenyi Biotec). The cell suspensions were filtered through a 70 μM-diameter cell strainer. Red blood cells were lysed using lysis buffer (Red Blood Cell Lysis Buffer, Biosharp), and dead cells were removed using the Dead Cell Removal Kit (Miltenyi Biotec). Finally, CD4^+^CD25^+^ Tregs were magnetically isolated using a CD4^+^CD25^+^ Regulatory T Cell Isolation Kit (Miltenyi Biotec).

### Flow cytometry analysis

Single cell suspensions were blocked at 4 ℃ for 30 min using 1 μg/ml mFc Block (BD, RRID: AB_394656) to prevent nonspecific antibody binding. Subsequently, the isolated cells were incubated at 4 ℃ for 30 min with various fluorescence-labeled antibodies and their corresponding isotype controls for surface staining. Following two washes, the cells were fixed and permeabilized using the Fixation and Permeabilization Solution (BD Biosciences, #554,722). After two washes, the cells were stained with fluorescence-labeled antibodies for 30 min for intracellular staining. Finally, the stained cells were washed and resuspended in 100 μL wash buffer before being evaluated using a flow cytometer (CYTOFLEX, Beckman). Data were analyzed with FlowJo_V10 software. The antibodies used in the experiment are listed in Table S7.

### Antibody binding assay

The affinity of anti-CCR8 antibodies was evaluated using flow cytometry. Briefly, Approximately 5 × 10^5^ 293 T cells stably overexpressing CCR8 (CCR8-293 T) were blocked with Human TruStain FcX™ (422,302, Biolegend) before being incubated with the test antibodies diluted with PBS/0.5% BSA (1:3 series dilution from 225 μg/ml to 0.00381 μg/ml) on ice for 20–30 min. After washing with PBS/0.5% BSA, cells were centrifuged, and the cell pellets were incubated with a secondary anti-mouse IgG-FITC (115–545-003, Jackson) on ice for 30 min. After washing, the cell pellets were resuspended in PBS/0.5% BSA before being analyzed with CYTOFLEX (Beckman). The binding affinity was calculated using GraphPad Prism.

### Chemotaxis assay

The chemotaxis of Tregs was assessed using a Transwell (Corning). Briefly, the migration medium containing 100 ng/ml of mCCL1 was added to the lower chamber of the 5.0 μm specification 96-well polycarbonate membrane HTS migration plate with 100 μL/well. Meanwhile, CCR8^+^ Tregs isolated from tumors were resuspended, adjusted the cell density of 1 × 10^6^ cells/mL with cell migration medium, placed into the upper chamber of the migration plate, with a volume of 100 μL/well, and incubated at 37 °C for 4 h. Afterwards, cells migrated to the lower chamber were counted using flow cytometer.

### Treg-CD8^+^ T cell co-culture

Tregs isolated from tumor tissues were seeded on plates containing 50 µL Dynabeads® mouse T-Activator CD3/CD28 (Gibco™) supplemented with murine IL2 (2000 U/mL). Meanwhile, Fresh CD8^+^ T cells isolated from spleens of healthy mice with CD8^+^ T Cell Isolation Kit (Miltenyi Biotec) were labeled with CFSE (Invitrogen™) and incubated with Tregs in a 1:1 ratio in the presence of murine CCL1 (100 ng/ml) for 3 days. The cells were harvested and analyzed using a flow cytometer with 488 nm excitation to measure the proliferation of CD8^+^ T cells. All the in vitro assays were conducted in triplicate, with three repeats.

### Real-Time PCR

Total RNA was extracted from tissues or cells using TRIzol (Life Technologies), and mRNA was polyadenylated using a poly-A polymerase-based First-Strand Synthesis kit (TaKaRa, Shanghai, China), reverse transcripted with a PrimeScript RT Reagent kit (TaKaRa), and cDNA was amplified and quantified using QuantStudio™ 6 Flex Real-Time PCR System (Applied Biosystems, Shanghai, China) and SYBR Green I (Applied Biosystems), respectively. The primers used in the reactions are listed in Table S6. For template, 1 mg of RNA was used for reverse transcription. The resulting cDNA was diluted 1/10 in ddH2O which accounts for 100 ng RNA per reaction. Analysis of each gene per samples was run in triplicate. The number of cycles was set to 40. Glyceraldehyde-3-phosphate dehydrogenase (GAPDH) was used as the endogenous control. Relative fold changes in the specific mRNA expression were calculated with the comparative threshold cycle (2^−ΔΔCt^) method.

### Bulk RNA sequencing

Total RNA was extracted from the indicated samples, and its integrity was assessed using the RNA Nano 6000 Assay Kit (Agilent Technologies, CA, USA, 5067–1511). The mRNA library was constructed with Novogene following standard operating procedures. The clustering of the index-coded samples was performed on a cBot Cluster Generation System using TruSeq PE Cluster Kit v3-cBot-HS (Illumia). After cluster generation, the library preparations were sequenced on an Illumina Novaseq platform, and 150 bp paired-end reads were generated. The detailed procedure is included in the Supplementary Protocol.

### Single-cell RNA sequencing

This study was performed by experienced personnel in the laboratory of NovelBio Co., Ltd. Tumor tissues were surgically removed and kept in magnetic-activated cell sorting solution (Miltenyi Biotec) before being washed with phosphate-buffered saline (PBS), minced into small pieces (approximately 1 mm^3^) on ice, and enzymatically digested with 1 mg/mL collagenase I (Worthington) and 200 μg/mL DNase I (Worthington) at 37 °C for 45 min, with agitation. Then, samples were sieved through a 70 µm cell strainer and centrifuged at 300 g for 5 min. After removing the supernatant, the pelleted cells were suspended in red blood cell lysis buffer (Miltenyi Biotec), and then washed with PBS containing 0.04% BSA. The cell pellets were resuspended in PBS containing 0.04% BSA and re-filtered through a 35 μm cell strainer. Cells were dissociated and subsequently stained with AO/PI for viability assessment using Countstar Fluorescence Cell Analyzer. The single-cell suspension was further enriched with a MACS dead cell removal kit (Miltenyi Biotec).

The single cell RNA sequencing (scRNA-Seq) libraries were generated using the 10 × Genomics Chromium Controller Instrument and Chromium Single Cell 3' V3.1 Reagent Kits (10 × Genomics, Pleasanton, CA). Briefly, cells were concentrated to approximately 1000 cells/μL and loaded into each channel to generate single-cell gel bead-in-emulsions (GEMs). After the RT step, GEMs were broken, and barcoded cDNA was purified and amplified. The amplified barcoded cDNA was fragmented, A-tailed, ligated with adaptors, and index PCR amplified. The final libraries were quantified using the Qubit High Sensitivity DNA assay (Thermo Fisher Scientific), and the size distribution of the libraries was determined using a High Sensitivity DNA chip on a Bioanalyzer 2200 (Agilent). All libraries were sequenced by Illumina sequencer (Illumina, San Diego, CA) on a 150 bp paired-end run. The detailed procedure of single-cell RNA statistical analysis is included in the Supplementary Protocol.

### Statistical analysis

Data were presented as means ± SD. Statistical analysis was performed using Student's t-test or One-way ANOVA. The relationship between CCR8 expression and clinical-pathological characteristics was analyzed using a chi-square test. Survival curves were plotted using the Kaplan–Meier method, and survival time was compared using a log-rank test. *p* < 0.05 was considered statistical significance.

## Results

### High infiltration of CCR8^+^ cells in tumor tissues of HCC patients is associated with poor prognosis

**Fig. 1:**
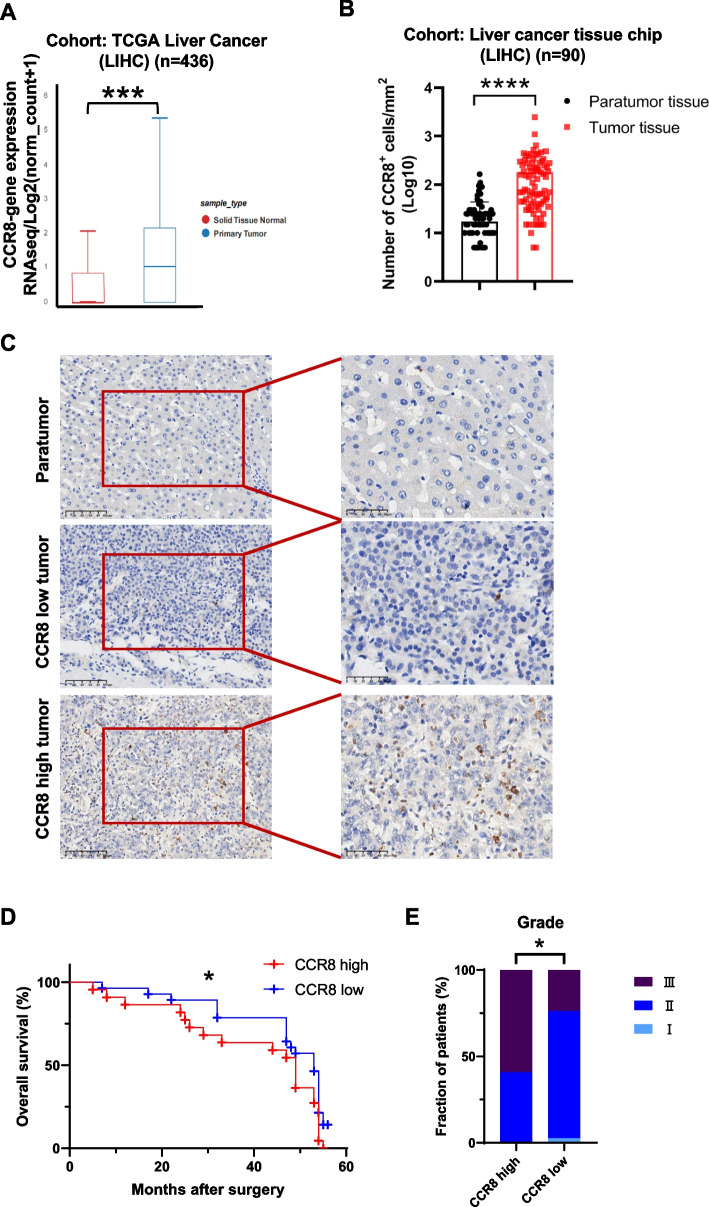
CCR8 is highly expressed in tumor tissues of HCC patients and associated with poor prognosis. **A** The expression level of CCR8 in primary tumor tissues and normal tissue from TCGA. **B** Quantification of CCR8 positive cells between the tumor tissues and corresponding paratumor tissues of HCC patients (*n* = 90). **C** The expression of CCR8 in paratumor tissues and tumor tissues from each HCC group was assessed by IHC analysis. Scale bar: 50 μm. **D** The Kaplan–Meier survival analysis plot showing a significant association between CCR8 expression and overall survival of HCC patients. **E** The proportion of different grades of HCC patients in CCR8 high and low groups. Data were shown as mean ± SD. *p < 0.05, ***p < 0.001, ****p < 0.0001, compared to control

Infiltration of CCR8^+^ Tregs in tumor tissue has been shown as a hallmark of many cancer types, with no report on HCC. To explore the potential involvement of CCR8 in the pathogenesis of HCC, we first compared the expression of CCR8 between HCC tissues and the adjacent normal solid tissues, also referred to as paratumor tissues. Data was retrieved from 436 samples in the liver cancer (Liver Hepatocellular Carcinoma, LIHC) cohort of The Cancer Genome Atlas Program (TCGA). The results indicated a positive relationship between the expression of *CCR8* and *Foxp3* (Fig. S1A) and a higher level of *CCR8* in primary tumor tissues relative to that in the adjacent normal tissue (Fig. 1A). To verify the TCGA results, immunohistochemistry (IHC) analysis was performed on HCC tissues (*n* = 90) and paired paratumor tissues (*n* = 90). As expected, a significantly higher infiltration of CCR8^+^ cells was observed in the HCC tissues compared to that in the paratumor tissues (Fig. 1B). It should be noted that, the expression level of CCR8 is not even in the tumor tissues, with relatively higher expression in some tumor tissues, and lower in some other tissues (Fig. 1C). Interestingly, the expression level of CCR8 appeared to be inversely correlated with survival rate, the higher the CCR8 expression, the lower the survival rate, as shown in the Kaplan–Meier survival analysis (Fig. 1D). Moreover, the expression of CCR8 was positively correlated with the malignant grade of the tumors (Fig. 1E). The IHC results further demonstrated the more the CCR8^+^ cell infiltration, the less the presence of CD45^+^ immune cells, CD4^+^ cells, CD8^+^ cells, as well as CD56^+^ cells in the tumor tissues (Fig. S1B-C). More confirmative results were included in the supplementary data section (Fig. S1D-E).

### CCR8 blockade suppressed liver cancer growth in syngeneic mouse model

**Fig. 2:**
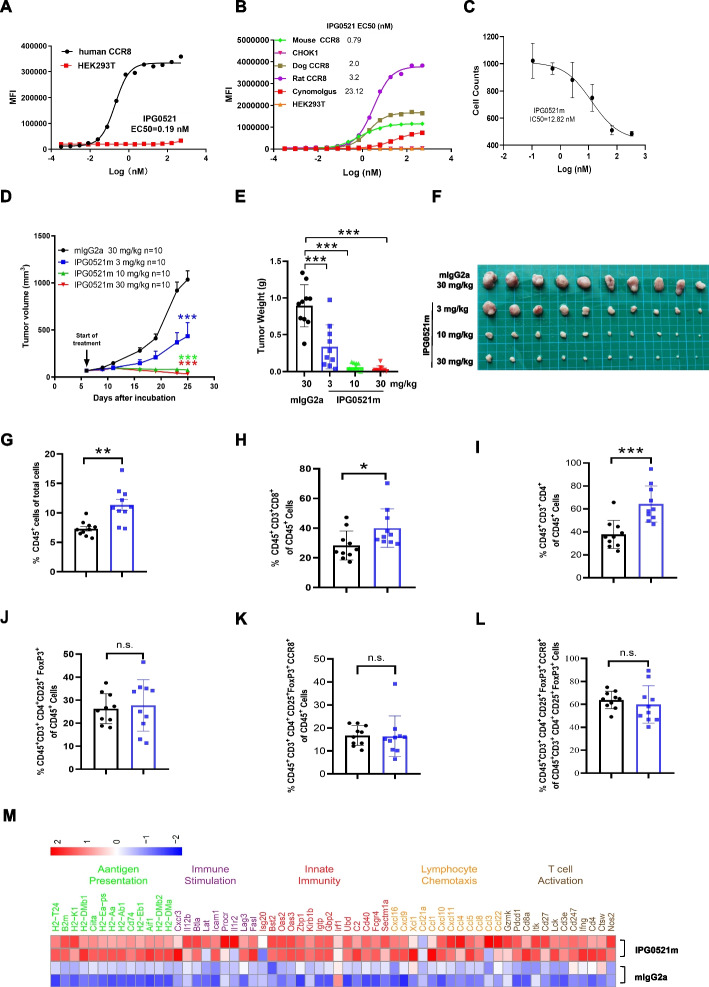
The CCR8 antagonist IPG0521 suppresses murine liver cancer growth via elevating CD8^+^ T cells without Treg reduction or depletion. **A** Binding activity of IPG0521 on CCR8-293 T with FCM analysis. **B** The specific binding of IPG0521 with mouse CCR8-CHOK1, rat CCR8-293 T, dog CCR8-293 T, and cynomolgus CCR8-293 T. Parental HEK293 cells were used as control. **C** Representative inhibition of IPG0521m in mCCL1 (100 ng/ml) mediated chemotaxis of Treg cells isolated from the tumor tissues of H22 syngeneic liver cancer model. **D** Dose-dependent inhibition of the syngeneic liver cancer growth in response to IPG0521m treatment. P values were calculated with tumor volume on day 19 after grouping using Tukey's multiple comparisons test. **E–F** Changes in tumor weight and size in response to different doses of IPG0521m administration. **G-L** Proportions of tumor-infiltrating CD45^+^ cells (**G**), CD8^+^ T cells (**H**), CD4^+^ T cells (**I**), total Tregs (**J**), CCR8^+^ Tregs in total CD45^+^ cells (**K**), and CCR8^+^ Tregs in total Tregs (**L**) with or without IPG0521m treatment (3 mg/kg). **M** RNA-seq analysis showing major changes in gene expression with or without IPG0521m treatment (3 mg/kg). Data were shown as mean ± SD. *p < 0.05, **p < 0.01, ***p < 0.001, compared to control

Previous studies have shown that CCR8 plays a role in the immunosuppressive function of Tregs [19], and antagonism of CCR8 has been reported to inhibit tumor growth in a few cancer types, including triple-negative breast cancer and bladder cancer [20, 21]. To examine whether CCR8 blockade suppresses liver cancer growth, we generated a monoclonal CCR8 antibody (Fig. S2-3, Table S1-3) named IPG0521, which bound CCR8 originated from human, monkey, dog, rat, and mouse with high affinity, and potently inhibited CCR8-mediated chemotaxis (Fig. 2A-C) and intracellular signaling (Fig. S3G-H). The specificity, structure, molecular weight, disulfide bonds, and glycosylation of IPG0521 were characterized (Fig. S4). Epitope mapping indicated that the antibody bound CCR8 at the second extracellular domain (EDC2), which possesses over 90% homology among the five species (Table S4), and the major binding site was aspartic acid 97, mutation of which resulted in over 100 times decrease in binding affinity (Fig. S3I and Table S5). As far as we know, this is so far the first CCR8 blocking antibody that is cross-reactive with human, mouse, and several other species. It is worth mentioning that IPG0521 has a stronger affinity than some other commercial products (Fig. S3J-O). In the following study involving a murine liver cancer model, IPG0521 with murine IgG2a Fc was constructed, named IPG0521m, which potently blocked the chemotaxis of CCR8^+^ Treg derived from murine cancer models (Fig. S5A-B).

To explore the antitumor effect of IPG0521m, a total of 40 mice was inoculated with H22 cells, a murine liver cancer cell line, randomly divided into 4 groups based on average tumor volume, and administrated with IPG0521m or murine mIgG2a isotype (control) twice a week for 19 days, during which, tumor volume was measured in every 2–3 days, and tumor growth inhibition rate was calculated. We observed that IPG0521m treatment resulted in a dose-dependent inhibition of tumor growth and complete tumor growth inhibition was observed at the dose of 3 mg/kg or above (Fig. 2D). The anticancer effect of IPG0521m was confirmed by measuring the tumor weight at the end of the study (Fig. 2E-F).

To understand the underlying mechanism, tumor infiltrated immune cells isolated from the IPG0521m (3 mg/kg), and isotype treatment groups were subjected to flow cytometry analysis. Total CD45^+^ cells were increased in response to IPG0521m treatment (Fig. 2G). Among the CD45^+^ cells, CD4^+^ T cell and CD8^+^ T cells were markedly elevated in the IPG0521m treatment group compared to that in the control group (Fig. 2H-I). Though total CD4^+^ T cells were increased upon the IPG0521m treatment, neither total Tregs nor CCR8^+^ Tregs were changed (Fig. 2I-L). IHC staining of CD45^+^ cells, CD4^+^ T cell, CD8^+^ T cells, FoxP3^+^ cells and NKP46^+^ cells provided more intuitive presentation for the change of immune cells after IPG0521m treatment (Fig. S6A-B). The anticancer immunity-boosting effect of IPG0521m was further confirmed with Bulk RNA-seq analysis, which indicated a marked increase in the expression of genes involved in antigen presentation, immune stimulation, innate immunity, lymphocyte chemotaxis, and T cell activation, in response to IPG0521m treatment (Fig. 2M). Similar results were obtained in the studies using HepG2 liver cancer model and murine lung cancer model (Fig. S6C-D).

### IPG0521m altered immune cell populations in the TIME of liver cancer

**Fig. 3:**
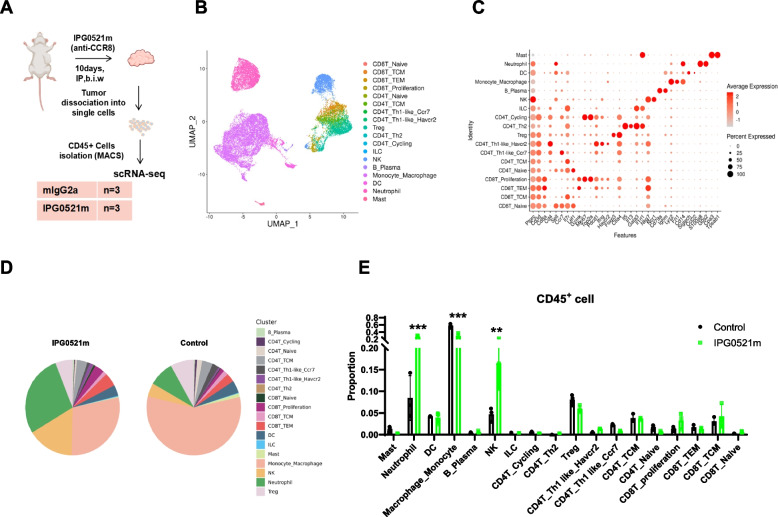
Landscape of CD45^+^ immune cells in response to IPG0521m treatment. **A** The scheme of the single cell RNA sequencing. **B** The Uniform Manifold Approximation and Projection (UMAP) of 10 × -based tumor infiltrated CD45^+^ immune cells single-cell data from 6 mice of the IPG0521m administration and control group showing 18 clusters in TIME identified by the integrated analysis, colored by cell cluster. Each single dot corresponds to one single cell colored according to cell cluster. **C** Gene bubble plot showing the expression of well-recognized marker genes in the major cell types. **D** Pie plot of cell cluster distribution in the IPG0521m administration group and control group, respectively. **E** The proportion of cell clusters in tumor infiltrated CD45^+^ immune cells in response to IPG0521m treatment. Data were shown as mean ± SD. **p < 0.01, ***p < 0.001, compare to control

The unexpected result that IPG0521m enhanced anticancer immunity without altering the Treg population in the TIME inspired us to explore the in-depth cellular mechanism using single-cell RNA sequencing (scRNA-seq). CD45^+^ cells sorted out from tumor tissues of H22 liver cancer bearing mice treated with IPG0521m or mIgG2a (control) were subjected to scRNA-seq (Fig. 3A). A total of 18 cell clusters were identified based on known cell lineage-specific marker genes unique to natural killer (NK) cells, neutrophils, dendritic cells (DCs), macrophages/monocytes, CD8^+^ T cells, CD4^+^ T cells, Tregs, ILC, mast cells, and B/plasma cells (Fig. 3B-C). As shown in Fig. 3D-E, NK cells and neutrophils were markedly increased, whereas macrophages/monocytes were significantly reduced in response to IPG0521m treatment, with no significant change in the percentage of Tregs, CD4^+^ T cells, CD8^+^ T cells, DCs, etc..

### IPG0521m treatment blunted the immunosuppressive effect of the tumor-infiltrating Tregs

**Fig. 4:**
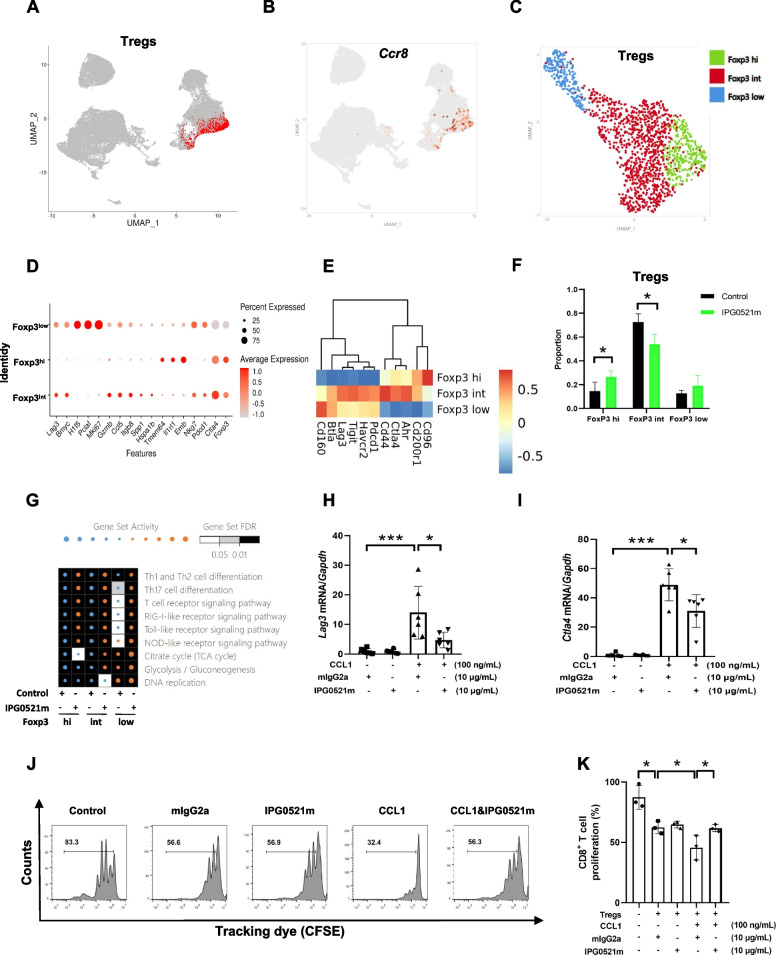
Effects of IPG0521m treatment on the phenotypes of TI-Tregs. **A** Distribution of Treg cells. **B** Expression of Ccr8 by UMAP plot. **C** UMAP projection of Treg cells, showing the formation of 3 subclusters (Foxp3^hi^, Foxp3^int^ and Foxp3^low^). Each dot represents an individual cell, colored according to cell cluster number. **D** The bubble plot shows the enrichment of distinct markers and Treg-related signature genes of different Treg subclusters. The bubble size represents the percentage of genes in the gene signature (x-axis) expressed in the corresponding cell subclusters (y-axis), and the color bar represents the average expression of genes. **E** The heat map showing the expression of immunosuppressive genes in Treg cell populations. **F** The cell proportion of Treg subclusters with or without IPG0521m administration based on the analysis of t-test. **G** Heat map showing the enrichment of pathways in Treg subclusters. **H-I** Tregs isolated from H22 liver cancer tissues were treated with mCCL1 (100 ng/ml) and IPG0521m (10 ug/ml) as indicated, and the expression of *Lag3* and *Ctla4* was evaluated using real-time PCR. **J** CFSE labeled CD8^+^ T cells isolated from spleens of healthy mice were co-cultured with Tregs isolated from H22 liver cancer tissues in the presence or absence of mCCL1 (100 ng/ml) and IPG0521m (10 ug/ml), and the proliferation of CD8^+^ T cells was assessed by means of FCM. **K** Quantification of CD8^+^ T cells proliferation described in (**J**). Data were shown as mean ± SD. *p < 0.05, ***p < 0.001

To explore whether IPG0521m treatment alters the intrinsic property and phenotype of the tumor-infiltrating Tregs (TI-Tregs), further clustering of Treg cell subsets was performed. The co-localization of CCR8^+^ cells with TI-Tregs indicated a predominant expression of CCR8 on TI-Tregs (Fig. 4A and B). According to the expression of FoxP3, TI-Tregs were classified into three clusters, namely high FoxP3 expression (Treg-FoxP3^hi^), low FoxP3 expression (Treg-FoxP3^low^), and intermediate FoxP3 expression (Treg-FoxP3^int^) (Fig. 4C). Interestingly, Treg-FoxP3^hi^ expressed a high level of lineage‐specific markers and a low level of immunosuppressive genes, such as *Pdcd1, Havcr2, Tigit, Lag3, Btla,* and *CD160*. In contrast, Treg-FoxP3^low^ expressed a relatively higher level of immunosuppressive genes and Mki67, suggesting that Treg-FoxP3^low^ cells are immunosuppressive subtype and highly proliferative (Fig. 4D-E). Strikingly, Treg-FoxP3^int^ cells, the largest proportion of TI-Tregs (Fig. 4C), also expressed a markedly higher level of immunosuppressive genes, with equivalent expression of Mki67, compared to the Treg-FoxP3^hi^ cells (Fig. 4D-E), suggesting that Treg-FoxP3^int^ cells are major immunosuppressive subtype of TI-Tregs. Interestingly, IPG0521m treatment resulted in a marked increase in the proportion of Treg-FoxP3^hi^ cells, with a dramatic decrease in the proportion of Treg-FoxP3^int^ cells (Fig. 4F). Quantitative Set Analysis for Gene Expression (QuSAGE) of all Treg sub-clusters showed that multiple immune-related pathways were activated in Tregs of IPG0521 administration group, compared with the control group (Fig. 4G). These data suggest that IPG0521m treatment resulted in a switch of TI-tregs phenotype from highly immunosuppressive to less immunosuppressive.

To confirm these findings, TI-Tregs isolated from H22 liver cancer tissues were incubated with murine CCL1 in the presence or absence of IPG0521m, and the expression of *Lag3* and *Ctla4* was measured. As expected, CCL1 stimulation of the TI-Tregs induced up-regulation of *Lag3* and *Ctla4*, which was reversed by IPG0521m treatment (Fig. 4H-I). To further confirm that the immunosuppressive function of TI-tregs is modulated by CCR8, CD8^+^ T cells labeled with carboxyfluorescein succinimidyl ester (CFSE) were co-cultured with TI-Tregs isolated from H22 liver cancer tissues, and after incubation of the co-culture with murine CCL1 in the presence or absence of IPG0521m, CD8^+^ T cell proliferation was measured. We observed that ligand-induced activation of CCR8 remarkably enhanced TI-tregs immunosuppression, leading to a dramatic reduction of CD8^+^ T cell proliferation, which was revered by IPG0521m (Fig. 4J-K).

### IPG0521m treatment enhanced the proliferation and cytotoxicity of CD8^+^ T cells and NK cells and promoted the activation of dendritic cells (DCs)

**Fig. 5:**
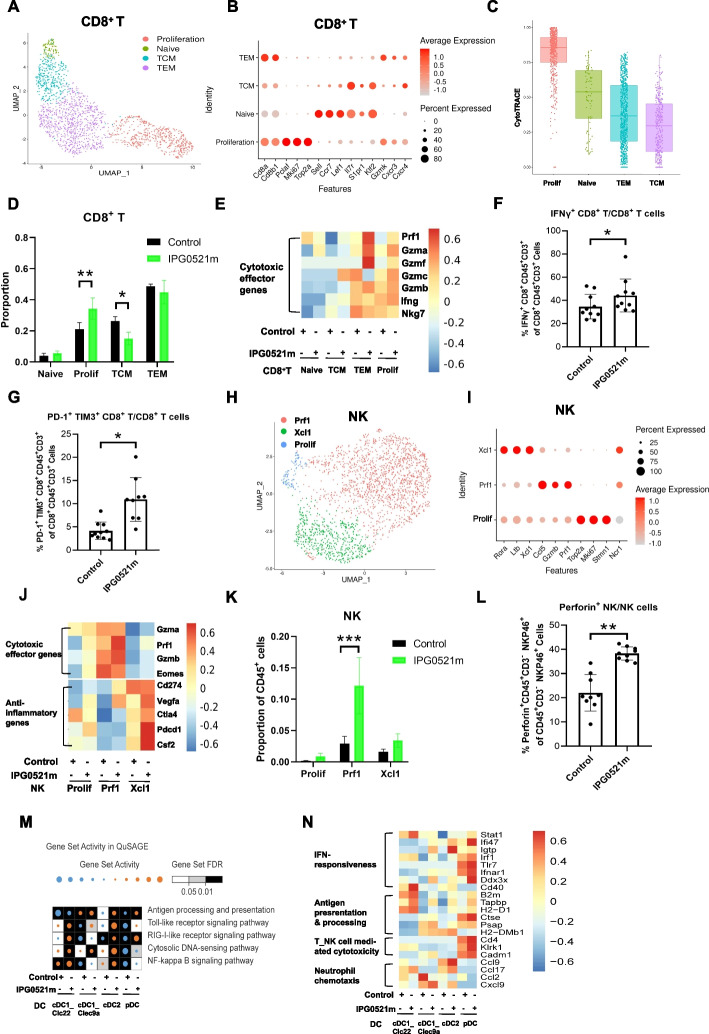
Effects of IPG0521m treatment on tumor-infiltrating CD8^+^ T cell, NK cells, and DCs. **A** UMAP projection of CD8^+^ T cells with 4 clusters. **B** The bubble plot shows the enrichment of distinct markers and CD8^+^ T cell-related signature genes of different CD8^+^ T cell subclusters. **C** Evaluation of the cytotoxicity of different CD8^+^ T cell subclusters using CytoTRACE analysis. **D** The proportional changes of CD8^+^ T cell subclusters in response to IPG0521m treatment. **E** The heat map indicating the changes of cytotoxicity-related gene expression in different CD8^+^ T cell subclusters in response to IPG0521m treatment. **F-G** Confirmation of the proportional changes in IFN-γ^+^ CD8^+^ T cells (**F**) and PD-1^+^ TIM3^+^ CD8^+^ T cells (**G**) by FCM analysis in response to IPG0521m treatment. **H** UMAP projection of NK cell clusters. **I** The bubble plot showing the enrichment of distinct markers and NK cell-related signature genes of different NK cell subclusters. **J** The heat map indicating changes of cytotoxicity-related gene and expression of anti-inflammatory genes in different NK cell subclusters in response to IPG0521m treatment. **K** The proportional changes of NK cell subclusters in response to IPG0521m treatment. **L** Confirmation of the proportional changes in perforin^+^ NK cells using FCM analysis in response to IPG0521m treatment. **M** Heat map showing the enrichment of pathways based on the QuSAGE of all DC subclusters. **N** The heat map showing the changes of gene expression related to different functions indicated in NK cells in response to IPG0521m treatment. Data were shown as mean ± SD. *p < 0.05, **p < 0.01, ***p < 0.001, compared to the control

The main function of Treg in TIME is to inhibit the anticancer immunity mediated by CD8^+^ T cells, NK cells, and DCs [6]. Based on the findings that IPG0521m abrogated the immunosuppressive function of TI-Tregs, we attempted to examine the intrinsic properties and potential functions of the CD8^**+**^ T cells, NK cells, and DCs following IPG0521m treatment of the H22 liver cancer mouse model. CD8^+^ T cells were classified into 4 clusters, including naïve, central memory (TCM), effector memory (TEM), and proliferative cells (Fig. 5A-C). The proliferative subset of CD8^**+**^ T cells, demonstrating the highest potency of cytotoxicity as determined by CytroTRACE analysis, were significantly increased after the administration of IPG0521m, concomitant with a decrease in the TCM group (Fig. 5D). Moreover, the expression of perforin and granzymes, which are essential for CD8^+^ T cell cytotoxicity, was elevated in response to IPG0521m treatment (Fig. 5E). As a confirmative study, flow cytometry analysis indicated a marked increase in IFN-γ^+^ CD8^+^ T cells upon IPG0521m treatment, though the population of PD-1^+^ TIM3^+^ CD8^+^ T cells was increased synchronously (Fig. 5F-G). Albeit the PD-1 + TIM3 + CD8 + T cells are postulated as exhausted CD8 + T cells, these cells are also associated with the activation of CD8 + T cells [22–24]. More in-depth investigations are needed to scrutinize their functions, but are beyond the scope the current study.

Three sub-clusters of NK cells were characterized, including NK cells expressing high levels of perforin (NK_Prf1), proliferative NK cells (NK_Prolif), and NK cells expressing the chemokine Xcl1 (NK_Xcl1) (Fig. 5H-I). The largest proportion was NK_Prf1 (Fig. 5H), the major cytotoxic subtype of NK cells with low anti-inflammatory gene expression. In addition, a special group defined as NK_Xcl1 cells with anti-inflammatory properties was found, which expressed some inhibitory genes like the previously reported NK^tolerant^ subset with regulatory functions (Fig. 5J) [25]. Interestingly, IPG0521m treatment resulted in an increase of all these three subtypes of NK cells, with the most profound increase in NK_Prf1 cells (Fig. 5J-L), suggesting that IPG0521m enhances the cytotoxicity of NK cells. Meanwhile, a slight increase in the proportion of NK_Xcl1 cells, NK cells with anti-inflammatory properties, was observed in response to IPG0521m treatment (Fig. 5J-K). This may be a feedback regulation of the significantly elevated cytotoxic function of NK cells.

DCs, the major antigen presenting cells, were classified into four sub-clusters, including plasmacytoid DCs (pDC), conventional type 2 DCs (cDC2), conventional types 1 DCs expressing CCL22 (cDC1_Ccl22), and conventional types 1 DCs expressing clec9a (cDC1_clec9a) (Fig. S**7**A-B). Interestingly, IPG0521m treatment resulted in elevated expression of genes involved in antigen processing and presentation, signaling involved in inflammatory cytokine expression, and T cell and NK cell activation in all of the sub-clusters of DCs (Fig. 5M-N). These data suggest that IPG0521m promotes the antigen processing and presenting functions of DCs.

### IPG0521m treatment reduced myeloid-derived suppressor cells, switched macrophage phenotypes to M1, and increased anticancer neutrophil proportion in TIME

**Fig. 6:**
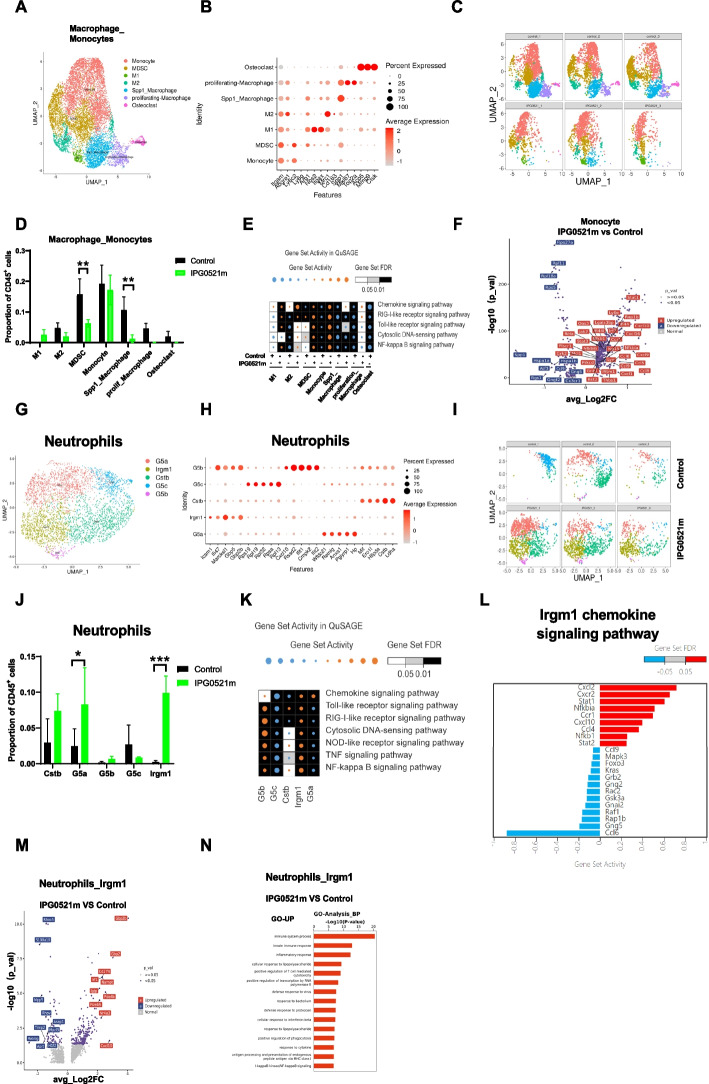
Effects of IPG0521m treatment on tumor-infiltrating monocytes, macrophages and neutrophils. **A** UMAP projection showing subclusters of tumor-infiltrating myeloid cells. **B** The bubble plot showing the enrichment of distinct markers of the tumor-infiltrating myeloid cell subclusters. **C** Individual UMAP projection showing the proportional changes in the tumor-infiltrating myeloid cell subclusters in response to IPG0521m treatment. **D** Quantification of the proportional changes in the tumor-infiltrating myeloid cell subclusters in response to IPG0521m treatment. **E** Heat map showing the enrichment of pathways in the tumor-infiltrating myeloid cell subclusters. **F** The volcano maps showing changes of differentially expressed genes in the tumor-infiltrating monocytes in response to IPG0521m treatment. **G** UMAP projection showing the tumor-infiltrating neutrophil subclusters. **H** The bubble plot showing the enrichment of distinct markers and neutrophil cell-related signature genes of different neutrophil subclusters. **I** Individual UMAP projection showing the proportional changes in the tumor-infiltrating neutrophil subclusters in response to IPG0521m treatment. **J** Quantification of the proportional changes in the tumor-infiltrating neutrophil subclusters in response to IPG0521m treatment. **K** Heat map showing the enrichment of pathways in neutrophil subclusters. **L** The GO analysis showing chemokine signaling pathways in Irgm1^+^ neutrophils. **M** The volcano maps showing changes in the differentially expressed genes (DEGs) in Irgm1^+^ neutrophils in response to IPG0521m treatment (**N**) The GO analysis showing signaling pathway changes in Irgm1^+^ neutrophils in response to IPG0521m treatment. Data were shown as mean ± SD. *p < 0.05, **p < 0.01, ***p < 0.001, compared to the control

Myeloid cells infiltrated in the tumor tissues were sub-clustered into seven phenotypes, including monocyte, myeloid-derived suppressor cells (MDSCs), M1 macrophages, M2 macrophages, macrophages expressing Spp1 (Spp1_macrophage), proliferative_macrophages, and Osteoclast-like cells (Fig. 6A-C). While monocytes were unchanged, MDSCs, the major immunosuppressive myeloid cells in TIME, were dramatically decreased in response to IPG0521m treatment (Fig. 6D). M2 macrophages, the immunosuppressive phenotype of macrophages, were reduced, and in contrast, M1 macrophages, the anti-inflammatory and antigen-presenting macrophages, were increased upon IPG0521m treatment. Spp1_macrophages, representing typical immunosuppressive macrophages that are critically involved in the progression of cancer [26–28], were reduced after IPG0521m treatment. Furthermore, proliferative macrophages and osteoclast-like macrophages were completely removed in response to IPG0521m treatment.

Along with the changes in myeloid cell proportion and phenotypes, chemokines and innate immunity signaling pathways, especially IFN-responsive genes, such as *STAT1, Ifi44, Irf7, Isg15*, and chemokines, including *CXCL1, CXCL9,* and *CXCL10*, were up-regulated in monocytes in response to IPG0521m treatment as assessed with Gene Set Activity in QuSAGE (Fig. 6E-F).

Tumor-associated neutrophils (TANs) were sub-clustered into five phenotypes, including G5b_, G5b_, Cstb_, Irgm1_, and G5a_neutrophils (Fig. 6G-H). Among these 5 subtypes, G5a_neutrophils, which demonstrate active chemokine signaling, were increased upon IPG0521m treatment (Fig. 6I-K). Furthermore, Irgm1_neutrophils, which exhibit highly active chemokine signaling pathways and innate immunity pathways (Fig. 6L), were significantly elevated in response to IPG0521m treatment (Fig. 6I-K).

### Depletion of CD8^+^ T cells partially abrogates the anti-tumor effect of IPG0521m

**Fig. 7:**
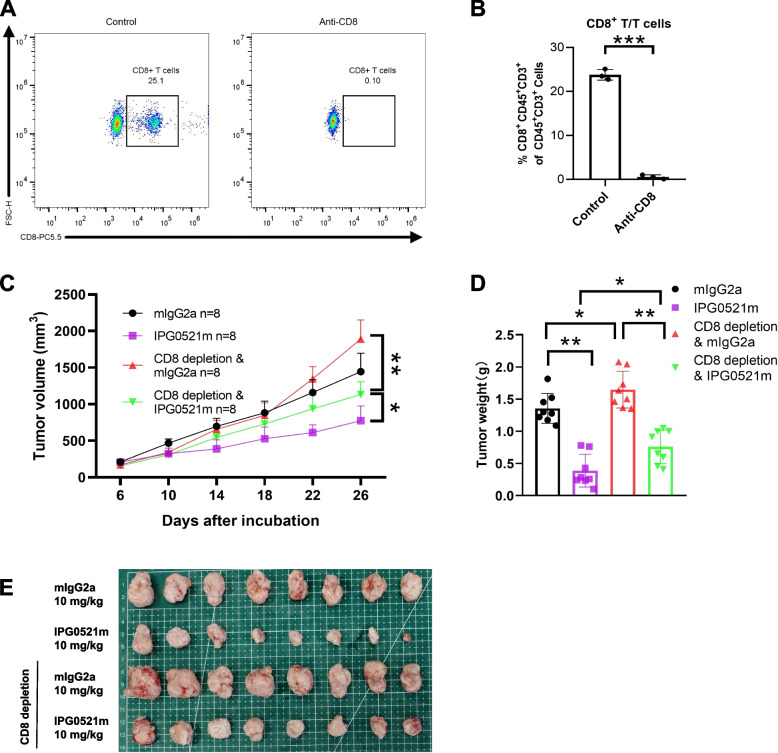
The anti-tumor function of IPG0521m is partially dependent on CD8^+^ T cells. **A** CD8^+^ T cell depletion was assessed by flow cytometry. **B** Quantification of CD8^+^ T cells proportion with or without CD8^+^ T cell depletion. **C** The growth curves of syngeneic liver cancer with or without CD8^+^ T cells depletion in response to IPG0521m treatment. **D**, **E** Changes in tumor weights (**D**) and tumor size (**E**) in each group. Data were shown as mean ± SD. *p < 0.05, **p < 0.01, ***p < 0.001, compared to the control

Given the specifically inhibitory of role of Tregs in CD8^+^ T cell cytotoxicity [29], we reasonably hypothesize that the anti-cancer effect of IPG0521m is attributable to its abrogation of Treg-mediated CD8^+^ T cell suppression. To confirm this hypothesis, we herein depleted CD8^+^ T cells using an anti-CD8α antibody, and examined the anti-cancer effect of IPG0521m. Successful CD8^+^ T cell depletion was verified by flow cytometry (Fig. 7A-B). Mice with or without CD8^+^ T cell depletion were inoculated with H22 tumor cells, followed by treatment with IPG0521m or mIgG2a. As expected, depletion of CD8^+^ T cells promoted tumor growth and weakened to a large extent the anti-cancer effect of IPG0521m (Fig. 7C-E). It should be noted that depletion of CD8^+^ T cells did not completely abolish the anti-cancer effect of IPG0521m, suggesting the involvement of other cell types, including, but not limited to, NK cells.

## Discussion

The immunosuppressive TME plays an important role in tumor escape of immune surveillance and contributes to the low remission rate and high frequency of resistance to immunotherapy. One of the major cell populations in TIME is Treg, which is fundamentally involved in the progression of HCC [30, 31]. Though the chemokine receptor CCR8 has been shown to be predominantly expressed on the surface of TI-Tregs, whether CCR8 is involved in the immunosuppressive functions of Treg remains controversial in the literature. In the present study, we demonstrated that ligand stimulation of CCR8^+^ Tregs enhanced the expression of genes critical for immunosuppression, which was reversed by IPG0521m. Treatment of liver cancer-bearing mice with IPG0521m resulted in a significant increase in CD8^+^ T cell infiltration and elevation of anticancer immunity. Though the proportion of TI-Tregs was unchanged, IPG0521m treatment resulted in a significant increase in TI-Tregs expressing low levels of immunosuppressive genes and a marked decrease in TI-Tregs expressing high levels of immunosuppressive genes, suggesting that CCR8 blockade blunted the immunosuppression of Tregs. These were associated with decreased MDSC and immunosuppressive macrophages, and increased cytotoxic NK cells, antigen-presenting DCs, as well as pro-inflammatory macrophages and neutrophils. Clearly, these data demonstrate that CCR8 blockade reserves the immunosuppressive TIME, resulting in a long-lasting anticancer immune response. Our results, in striking contrast to the anti-CCR8 ADCC strategy, pave the way for a safer and more effective treatment of HCC and other cancers.

With regard to the role of CCR8 in the functions of TI-Tregs, intense disputations occur in the literature. There are contradictory reports regarding the requirement of CCR8-mediated signaling for Treg immunosuppression. For instance, a previous study showed that tumor growth was not inhibited and TI-Tregs were not reduced in CCR8 knockout mice bearing murine colon cancer or melanoma, which led to the conclusion that CCR8 is merely a mark of the highly suppressive TI-Tregs but is dispensable for their accumulation and suppressive function [8]. It is argued that given the intricate and redundant nature of the chemokine system [32], deficiency of CCR8 during initial embryonic development may cause compensation of its role by other chemokine receptors, resulting unchanged Treg phenotype in the tumor-bearing animals. Another study showed that tumor inhibition was only achieved by means of cell-depleting anti-CCR8 with enhanced ADCC rather than CCR8 blocking antibody [13]. However, it is noted that the IC_50_ of the ADCC-deficient anti-CCR8 mAb is several times higher than that of ADCC-intact mAb in terms of signaling blockade [13]. Thus, it is deeply concerned whether this weak signal-blocking ADCC-deficient anti-CCR8 mAb could effectively modulate the TI-Tregs phenotype and exert anticancer efficacy in vivo. In contrast, IPG0521m, which bound murine CCR8 with high affinity and potently blocked CCR8-mediated signaling in different assays, including β-arrestin assay and chemotaxis assay, with a nanomolar range of IC50, was shown to inhibit tumor growth in a dose-dependent manner. Echoing our findings, several studies have shown the critical role of CCR8 in Treg immunosuppression. For instance, Barsheshet Y et al. reported that stimulation of CCR8^+^ Tregs with CCL1 resulted in enhanced suppressive activity of these cells [18]. In a gastric cancer mouse model, CCR8 blockade downregulated Treg-produced IL-10 and reversed the suppression by Tregs on the secretion and proliferation of CD8^+^ T cells [19]. In the muscle-invasive bladder cancer model, CCR8 blockade destabilized intratumoral Tregs into a fragile phenotype, associated with reactivation of antitumor immunity and augment of anti-PD-1 therapeutic benefit [20]. Nevertheless, it is now clarified that CCR8 is not merely a biomarker for TI-Tregs in the TME, but plays a crucial role in the immunosuppressive function of TI-Tregs.

We noted with great excitement that IPG0521m treatment did not cause reduction or depletion of TI-Tregs as observed in other studies using CCR8 monoclonal antibodies with enhanced ADCC functions [9, 12, 33]. To unveil the underlying mechanism, we performed scRNA-seq. It was clearly shown in response to IPG0521m treatment, the population of Tregs expressing high level of immunosuppressive genes declined, and instead, the population of Tregs expression low level of immunosuppressive genes increased, suggesting that IPG0521m blunted the immunosuppression of Tregs in the liver cancer-bearing mice. The scRNA-seq further demonstrated that along with the phenotypic change in Tregs, the CD8^+^ T cell proliferation and cytotoxicity were both increased, suggesting that the anti-cancer effect of IPG0521m is largely attributable to the abolishment of Treg-mediated inhibiton of CD8^+^ T cell cytotoxicity. This notion was confirmed by our study showing that depletion of CD8^+^ T cells abrogated to a large extent the anti-cancer effect of IPG0521m.

The mechanisms underlying the anticancer effect of IPG0521m appear to extend far beyond its modulation of the TI-Tregs phenotype. IPG0521 treatment resulted in dramatic changes in the population and phenotype of myeloid cells, which are comprised of MDSCs, macrophages, dendritic cells, monocytes, and granulocytes. MDSCs represent a major component of the TIME and are critically involved in the regulation of tumor progression and metastasis [34]. One of the intriguing findings was that MDSCs were markedly reduced in response to IPG0521m treatment. This is unlikely to be attributable to its direct regulation of MDSC since there is no evidence showing the expression of CCR8 on these cells. We postulate that the reduction in MDSC in response to IPG0521m treatment is likely due to the crosstalk between Tregs and MDSCs, both of which have been shown to collaborate with each other in promoting tumor-immune evasion [35–37], and both of which are able to induce generation and enhance the suppressive activity of the other via various signaling pathways, including PD-L1 signaling [38, 39].

Another interesting finding is that IPG0521m treatment induced a reduction of M2 macrophages and an increase in M1 macrophages, suggesting that TAMs undergo a switch from immunosuppressive to pro-inflammatory phenotype. As the major tumor-infiltrating immune cell population, TAMs are commonly educated by tumor cells to become their partners in crime, promoting tumor immune escape, angiogenesis, tumor growth, and metastasis [40, 41]. Notably, TAMs are phenotypically described as M2 macrophages that are alternatively activated by Th2 cytokines. By contrast, tumor-killing macrophages are typically described as M1 macrophages that are classically activated by Th1 cytokines [40, 42]. Therefore, converting M2 TAMs into M1 antitumor macrophages is an ideal approach to target tumor-infiltrating macrophages. Clearly, the IPG0521m-induced switch in the phenotypes of TAMs from M2 to M1 is one of the mechanisms accountable for its anticancer effect. It is unlikely that IPG0521m directly modulates the TAM phenotype as there is no report of the expression of CCR8 on TAMs. This phenotypic change in TAMs in response to IPG0521m treatment is likely attributable to the crosstalk between Tregs and TAMs that has been revealed elsewhere [43].

The third interesting finding is that IPG0521m treatment resulted in a marked increase in TANs, most notably IRGM1^+^ neutrophils, in TIME. Neutrophils act as the body’s first line of defense against infection and respond to diverse inflammatory cues, including cancer. Although the role of IRGM1 in neutrophils in cancer scenarios remains to be investigated, based on the recent study that the IRGM1 is required to promote T cell-mediated control of infection in neutrophils [44], and on our results that IRGM1^+^ neutrophils express high levels of CXCL2, CXCL10, and CXCR2, chemokines and receptors involved in neutrophil recruitment and T cell activation, we reasonably postulate that the IRGM1^+^ neutrophils promote anticancer immunity. Though it is largely unknown how CCR8 blockade with UIPG0521m resulted in increased infiltration of IRGM1^+^ neutrophils, a study with an inflammatory disease model indicated that CCR8 deficiency reduced eosinophil accumulation but accelerated neutrophil accumulation in the inflammatory site, suggesting that CCR8 blockade may promote neutrophil accumulation [45]. An in-depth investigation will be executed to explore the underlying mechanisms in the future.

Taken together, we provided solid evidence to illustrate that CCR8 is not merely a biomarker for Treg cells, but rather plays a crucial role in the immunosuppressive functions of TI-Tregs. CCR8 antagonism robustly suppressed liver cancer growth via switching the highly immunosuppressive TI-Tregs to less an immunosuppressive phenotype, leading to elevated anticancer immunity and a long-lasting tumor-suppressing effect. CCR8 antagonist may provide a much safer therapy for cancer relative to the anti-CCR8 ADCC strategy, the latter may cause severe side effects due to the expression of CCR8 not only on TI-Tregs but also on Th2 cells, Th17 cells, central memory CD4^+^ and CD8^+^ T cells, NKT cells, monocytes, vascular endothelial cells, and various normal tissues [14–17].

## Supplementary Information

Supplementary Material 1.

## Data Availability

Data are available upon reasonable request.

## Abbreviations

HCC: Hepatocellular carcinoma
TIME: Tumor immune microenvironment
Tregs: Regulatory T cells
TME: Tumor microenvironment
TI-Tregs: Tumor-infiltrating Tregs
TAMs: Tumor-associated macrophages
ADCC: Antibody dependent cell mediated cytotoxicity
MACS: Magnetic-activated cell sorting
RNA-seq: RNA-sequencing
scRNA-seq: Single-cell RNA sequencing
NK cells: Natural killer cells
DCs: Dendritic cells
MDSCs: Myeloid-derived suppressor cells
TANs: Tumor-associated neutrophils

## References

[1] Siegel RL, Miller KD, Fuchs HE, Jemal A. Cancer statistics, 2022. CA Cancer J Clin. 2022;72(1):7–33. 10.3322/caac.21708. 35020204 10.3322/caac.21708

[2] Ohue Y, Nishikawa H. Regulatory T (Treg) cells in cancer: Can Treg cells be a new therapeutic target? Cancer Sci. 2019;110(7):2080–9. 10.1111/cas.14069. 31102428 10.1111/cas.14069 PMC6609813

[3] Bai Y, Chen D, Cheng C, Li Z, Chi H, Zhang Y, et al. Immunosuppressive landscape in hepatocellular carcinoma revealed by single-cell sequencing. Front Immunol. 2022;13: 950536. 10.3389/fimmu.2022.950536. 35967424 10.3389/fimmu.2022.950536 PMC9365996

[4] Lan YT, Fan XP, Fan YC, Zhao J, Wang K. Change in the Treg/Th17 cell imbalance in hepatocellular carcinoma patients and its clinical value. Medicine (Baltimore). 2017;96(32): e7704. 10.1097/MD.0000000000007704. 28796055 10.1097/MD.0000000000007704 PMC5556221

[5] Wang L, Simons DL, Lu X, Tu TY, Solomon S, Wang R, et al. Connecting blood and intratumoral T(reg) cell activity in predicting future relapse in breast cancer. Nat Immunol. 2019;20(9):1220–30. 10.1038/s41590-019-0429-7. 31285626 10.1038/s41590-019-0429-7 PMC8802768

[6] Haruna M, Ueyama A, Yamamoto Y, Hirata M, Goto K, Yoshida H, et al. The impact of CCR8+ regulatory T cells on cytotoxic T cell function in human lung cancer. Sci Rep. 2022;12(1):5377. 10.1038/s41598-022-09458-5. 35354899 10.1038/s41598-022-09458-5 PMC8967908

[7] Villarreal D, L’Huillier A, Armington S, Mottershead C, Filippova E, Coder B, et al. Targeting CCR8 Induces Protective Antitumor Immunity and Enhances Vaccine-Induced Responses in Colon Cancer. Can Res. 2018;78(18):5340–8. 10.1158/0008-5472.can-18-1119. 10.1158/0008-5472.CAN-18-1119 30026324

[8] Whiteside SK, Grant FM, Gyori DS, Conti AG, Imianowski CJ, Kuo P, et al. CCR8 marks highly suppressive Treg cells within tumours but is dispensable for their accumulation and suppressive function. Immunology. 2021;163(4):512–20. 10.1111/imm.13337. 33838058 10.1111/imm.13337 PMC8274197

[9] Van Damme H, Dombrecht B, Kiss M, Roose H, Allen E, Van Overmeire E, et al. Therapeutic depletion of CCR8(+) tumor-infiltrating regulatory T cells elicits antitumor immunity and synergizes with anti-PD-1 therapy. J Immunother Cancer. 2021;9(2). 10.1136/jitc-2020-001749. 10.1136/jitc-2020-001749 PMC7887378 33589525

[10] Nagira Y, Nagira M, Nagai R, Nogami W, Hirata M, Ueyama A, et al. S-531011, a Novel Anti-Human CCR8 Antibody, Induces Potent Antitumor Responses through Depletion of Tumor-Infiltrating CCR8-Expressing Regulatory T Cells. Mol Cancer Ther. 2023;22(9):1063–72. 10.1158/1535-7163.MCT-22-0570. 37420296 10.1158/1535-7163.MCT-22-0570 PMC10477828

[11] Suzuki HI, Onimaru K. Biomolecular condensates in cancer biology. Cancer Sci. 2022;113(2):382–91. 10.1111/cas.15232. 34865286 10.1111/cas.15232 PMC8819300

[12] Weaver JD, Stack EC, Bugge JA, Hu C, McGrath L, Mueller A, et al. Differential expression of CCR8 in tumors versus normal tissue allows specific depletion of tumor-infiltrating T regulatory cells by GS-1811, a novel Fc-optimized anti-CCR8 antibody. Oncoimmunology. 2022;11(1):2141007. 10.1080/2162402X.2022.2141007. 36352891 10.1080/2162402X.2022.2141007 PMC9639568

[13] Kidani Y, Nogami W, Yasumizu Y, Kawashima A, Tanaka A, Sonoda Y, et al. CCR8-targeted specific depletion of clonally expanded Treg cells in tumor tissues evokes potent tumor immunity with long-lasting memory. Proc Natl Acad Sci U S A. 2022;119(7). 10.1073/pnas.2114282119. 10.1073/pnas.2114282119 PMC8851483 35140181

[14] Liu L, Doijen J, D’Huys T, Verhaegen Y, Dehaen W, De Jonghe S, et al. Biological characterization of ligands targeting the human CC chemokine receptor 8 (CCR8) reveals the biased signaling properties of small molecule agonists. Biochem Pharmacol. 2021;188: 114565. 10.1016/j.bcp.2021.114565. 33872569 10.1016/j.bcp.2021.114565

[15] Soler D, Chapman TR, Poisson LR, Wang L, Cote-Sierra J, Ryan M, et al. CCR8 expression identifies CD4 memory T cells enriched for FOXP3+ regulatory and Th2 effector lymphocytes. J Immunol. 2006;177(10):6940–51. 10.4049/jimmunol.177.10.6940. 17082609 10.4049/jimmunol.177.10.6940

[16] Tiffany HL, Lautens LL, Gao JL, Pease J, Locati M, Combadiere C, et al. Identification of CCR8: a human monocyte and thymus receptor for the CC chemokine I-309. J Exp Med. 1997;186(1):165–70. 10.1084/jem.186.1.165. 9207005 10.1084/jem.186.1.165 PMC2198957

[17] Liu X, Xu X, Deng W, Huang M, Wu Y, Zhou Z, et al. CCL18 enhances migration, invasion and EMT by binding CCR8 in bladder cancer cells. Mol Med Rep. 2019;19(3):1678–86. 10.3892/mmr.2018.9791. 30592282 10.3892/mmr.2018.9791 PMC6390063

[18] Barsheshet Y, Wildbaum G, Levy E, Vitenshtein A, Akinseye C, Griggs J, et al. CCR8(+)FOXp3(+) T(reg) cells as master drivers of immune regulation. Proc Natl Acad Sci U S A. 2017;114(23):6086–91. 10.1073/pnas.1621280114. 28533380 10.1073/pnas.1621280114 PMC5468670

[19] Zhang Z, Wang G, Shao X, Wu H, Su X, Zhu L, et al. A Novel Prognostic Biomarker CCR8 for Gastric Cancer and Anti-CCR8 Blockade Attenuate the Immunosuppressive Capacity of Tregs In Vitro. Cancer Biother Radiopharm. 2023;38(6):415–24. 10.1089/cbr.2022.0095. 37102694 10.1089/cbr.2022.0095

[20] Wang T, Zhou Q, Zeng H, Zhang H, Liu Z, Shao J, et al. CCR8 blockade primes anti-tumor immunity through intratumoral regulatory T cells destabilization in muscle-invasive bladder cancer. Cancer Immunol Immunother. 2020;69(9):1855–67. 10.1007/s00262-020-02583-y. 32367308 10.1007/s00262-020-02583-y PMC11027714

[21] Wu Y, Xi J, Li Y, Li Z, Zhang Y, Wang J, et al. Discovery of a Potent and Selective CCR8 Small Molecular Antagonist IPG7236 for the Treatment of Cancer. J Med Chem. 2023;66(7):4548–64. 10.1021/acs.jmedchem.3c00030. 36988587 10.1021/acs.jmedchem.3c00030

[22] Oestreich KJ, Yoon H, Ahmed R, Boss JM. NFATc1 regulates PD-1 expression upon T cell activation. J Immunol. 2008;181(7):4832–9. 10.4049/jimmunol.181.7.4832. 18802087 10.4049/jimmunol.181.7.4832 PMC2645436

[23] Zhang Z, Ren C, Xiao R, Ma S, Liu H, Dou Y, et al. Palmitoylation of TIM-3 promotes immune exhaustion and restrains antitumor immunity. Sci Immunol. 2024;9(101):eadp7302. 10.1126/sciimmunol.adp7302. 10.1126/sciimmunol.adp7302 39546589

[24] Xiong H, Mittman S, Rodriguez R, Pacheco-Sanchez P, Moskalenko M, Yang Y, et al. Coexpression of Inhibitory Receptors Enriches for Activated and Functional CD8(+) T Cells in Murine Syngeneic Tumor Models. Cancer Immunol Res. 2019;7(6):963–76. 10.1158/2326-6066.CIR-18-0750. 31064777 10.1158/2326-6066.CIR-18-0750

[25] Fu B, Tian Z, Wei H. Subsets of human natural killer cells and their regulatory effects. Immunology. 2014;141(4):483–9. 10.1111/imm.12224. 24303897 10.1111/imm.12224 PMC3956422

[26] Liu Y, Xun Z, Ma K, Liang S, Li X, Zhou S, et al. Identification of a tumour immune barrier in the HCC microenvironment that determines the efficacy of immunotherapy. J Hepatol. 2023;78(4):770–82. 10.1016/j.jhep.2023.01.011. 36708811 10.1016/j.jhep.2023.01.011

[27] Liu Y, Zhang Q, Xing B, Luo N, Gao R, Yu K, et al. Immune phenotypic linkage between colorectal cancer and liver metastasis. Cancer Cell. 2022;40(4):424–37 e5. 10.1016/j.ccell.2022.02.013. 10.1016/j.ccell.2022.02.013 35303421

[28] Matsubara E, Komohara Y, Esumi S, Shinchi Y, Ishizuka S, Mito R, et al. SPP1 Derived from Macrophages Is Associated with a Worse Clinical Course and Chemo-Resistance in Lung Adenocarcinoma. Cancers (Basel). 2022;14(18). 10.3390/cancers14184374. 10.3390/cancers14184374 PMC9496817 36139536

[29] Tanaka A, Sakaguchi S. Targeting Treg cells in cancer immunotherapy. Eur J Immunol. 2019;49(8):1140–6. 10.1002/eji.201847659. 31257581 10.1002/eji.201847659

[30] Kohli K, Pillarisetty VG, Kim TS. Key chemokines direct migration of immune cells in solid tumors. Cancer Gene Ther. 2022;29(1):10–21. 10.1038/s41417-021-00303-x. 33603130 10.1038/s41417-021-00303-x PMC8761573

[31] Gungabeesoon J, Gort-Freitas NA, Kiss M, Bolli E, Messemaker M, Siwicki M, et al. A neutrophil response linked to tumor control in immunotherapy. Cell. 2023;186(7):1448–64 e20. 10.1016/j.cell.2023.02.032. 10.1016/j.cell.2023.02.032 PMC10132778 37001504

[32] Dyer DP, Medina-Ruiz L, Bartolini R, Schuette F, Hughes CE, Pallas K, et al. Chemokine Receptor Redundancy and Specificity Are Context Dependent. Immunity. 2019;50(2):378–89 e5. 10.1016/j.immuni.2019.01.009. 10.1016/j.immuni.2019.01.009 PMC6382461 30784579

[33] Campbell JR, McDonald BR, Mesko PB, Siemers NO, Singh PB, Selby M, et al. Fc-Optimized Anti-CCR8 Antibody Depletes Regulatory T Cells in Human Tumor Models. Cancer Res. 2021;81(11):2983–94. 10.1158/0008-5472.CAN-20-3585. 33757978 10.1158/0008-5472.CAN-20-3585

[34] van Vlerken-Ysla L, Tyurina YY, Kagan VE, Gabrilovich DI. Functional states of myeloid cells in cancer. Cancer Cell. 2023;41(3):490–504. 10.1016/j.ccell.2023.02.009. 36868224 10.1016/j.ccell.2023.02.009 PMC10023509

[35] Dannenmann SR, Thielicke J, Stockli M, Matter C, von Boehmer L, Cecconi V, et al. Tumor-associated macrophages subvert T-cell function and correlate with reduced survival in clear cell renal cell carcinoma. Oncoimmunology. 2013;2(3): e23562. 10.4161/onci.23562. 23687622 10.4161/onci.23562 PMC3655740

[36] Khaled YS, Ammori BJ, Elkord E. Myeloid-derived suppressor cells in cancer: recent progress and prospects. Immunol Cell Biol. 2013;91(8):493–502. 10.1038/icb.2013.29. 23797066 10.1038/icb.2013.29

[37] Haist M, Stege H, Grabbe S, Bros M. The Functional Crosstalk between Myeloid-Derived Suppressor Cells and Regulatory T Cells within the Immunosuppressive Tumor Microenvironment. Cancers (Basel). 2021;13(2). 10.3390/cancers13020210. 10.3390/cancers13020210 PMC7827203 33430105

[38] Fujimura T, Kambayashi Y, Aiba S. Crosstalk between regulatory T cells (Tregs) and myeloid derived suppressor cells (MDSCs) during melanoma growth. Oncoimmunology. 2012;1(8):1433–4. 10.4161/onci.21176. 23243619 10.4161/onci.21176 PMC3518528

[39] Tiemessen MM, Jagger AL, Evans HG, van Herwijnen MJ, John S, Taams LS. CD4+CD25+Foxp3+ regulatory T cells induce alternative activation of human monocytes/macrophages. Proc Natl Acad Sci U S A. 2007;104(49):19446–51. 10.1073/pnas.0706832104. 18042719 10.1073/pnas.0706832104 PMC2148309

[40] Chen D, Xie J, Fiskesund R, Dong W, Liang X, Lv J, et al. Chloroquine modulates antitumor immune response by resetting tumor-associated macrophages toward M1 phenotype. Nat Commun. 2018;9(1):873. 10.1038/s41467-018-03225-9. 29491374 10.1038/s41467-018-03225-9 PMC5830447

[41] De Henau O, Rausch M, Winkler D, Campesato LF, Liu C, Cymerman DH, et al. Overcoming resistance to checkpoint blockade therapy by targeting PI3Kgamma in myeloid cells. Nature. 2016;539(7629):443–7. 10.1038/nature20554. 27828943 10.1038/nature20554 PMC5634331

[42] Biswas SK, Mantovani A. Macrophage plasticity and interaction with lymphocyte subsets: cancer as a paradigm. Nat Immunol. 2010;11(10):889–96. 10.1038/ni.1937. 20856220 10.1038/ni.1937

[43] Huang L, Zhao Y, Shan M, Wang S, Chen J, Liu Z, et al. Targeting crosstalk of STAT3 between tumor-associated M2 macrophages and Tregs in colorectal cancer. Cancer Biol Ther. 2023;24(1):2226418. 10.1080/15384047.2023.2226418. 37381162 10.1080/15384047.2023.2226418 PMC10312030

[44] Naik SK, McNehlan ME, Mreyoud Y, Kinsella RL, Smirnov A, Chowdhury CS, et al. Type I IFN signaling in the absence of IRGM1 promotes M. tuberculosis replication in immune cells by suppressing T cell responses. bioRxiv. 2023. 10.1101/2023.10.03.560720. 10.1016/j.mucimm.2024.07.002 39038752

[45] Blanco-Perez F, Kato Y, Gonzalez-Menendez I, Laino J, Ohbayashi M, Burggraf M, et al. CCR8 leads to eosinophil migration and regulates neutrophil migration in murine allergic enteritis. Sci Rep. 2019;9(1):9608. 10.1038/s41598-019-45653-7. 31270368 10.1038/s41598-019-45653-7 PMC6610106

