# Supplementary material for: CCR8 antagonist suppresses liver cancer progression via turning tumor-infiltrating Tregs into less immunosuppressive phenotype
Source: J Exp Clin Cancer Res. 2025 Apr 4;44:113. doi: 10.1186/s13046-025-03286-x (PMC11969927; doi:10.1186/s13046-025-03286-x)
Supplement: Supplementary file 1 — Supplementary Material 1. [file 13046_2025_3286_MOESM1_ESM.doc]

**Supplementary Materials**

**Figs. S1 to S7 for multiple supplementary figures**


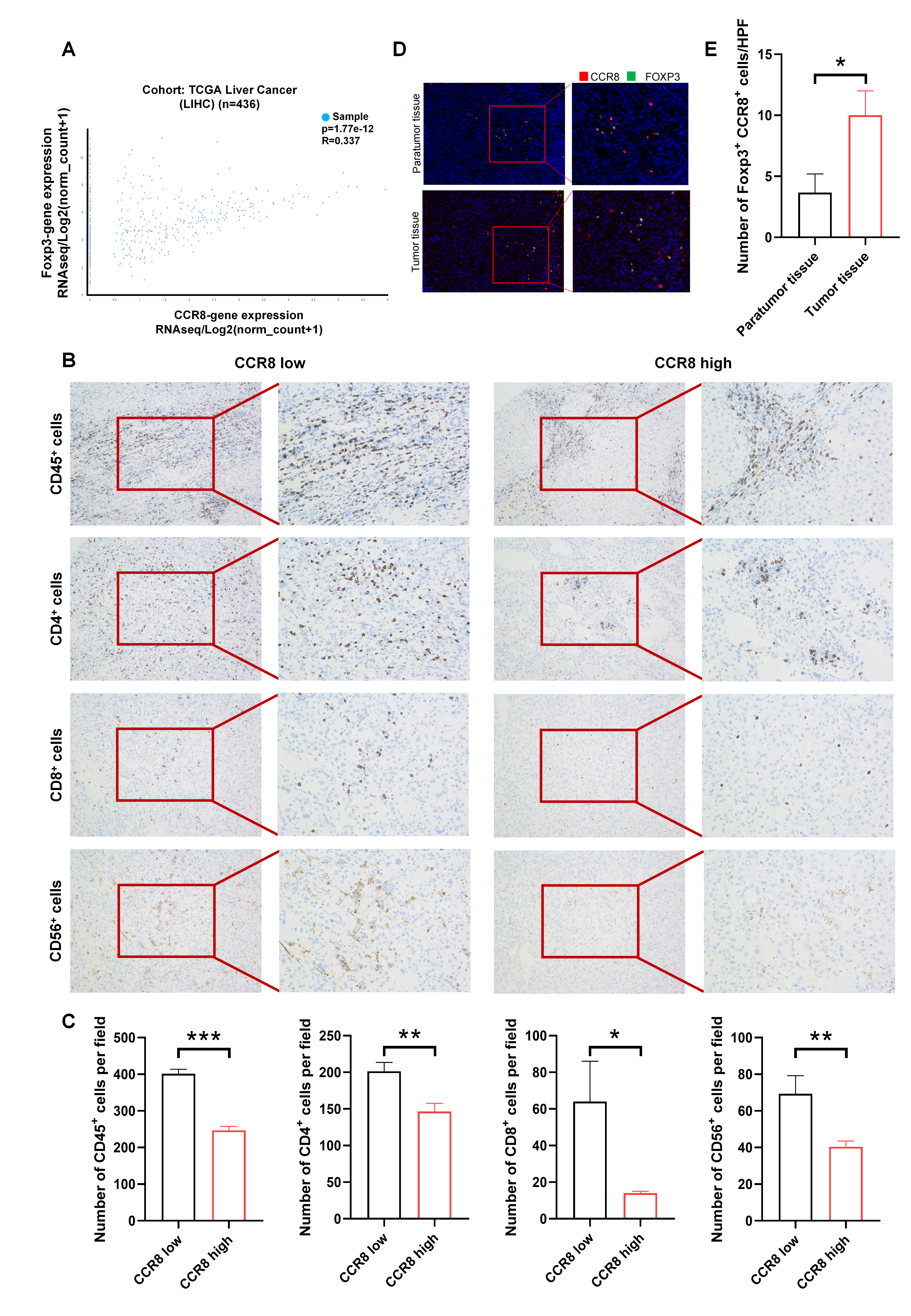
**Fig. S1. Co-expression of CCR8 and Foxp3 in HCC, and IHC analysis of immune cells in tumors with different CCR8 expression.**

**(A)** The positive correlation between the expression of CCR8 and Foxp3 in LIHC from TCGA. **(B)** The expression of CD45^+^ immune cells, CD8^+^ cells, CD4^+^ cells, as well as CD56^+^ cells in CCR8 high tumors and CCR8 low tumors was assessed by IHC analysis. **(C)** Quantification of these cells described in (B). **(D)** Representative immunofluorescent microscopy images showing CCR8^+^ Foxp3^+^ cells in the tumor tissues and corresponding paratumor tissues of HCC patients. **(E)** Quantification of CCR8^+^ Foxp3^+^ cells in the experiment described in (b). Data were shown as mean ± SD. ******p*＜ 0.05, *******p*＜ 0.01, ********p*＜ 0.001.


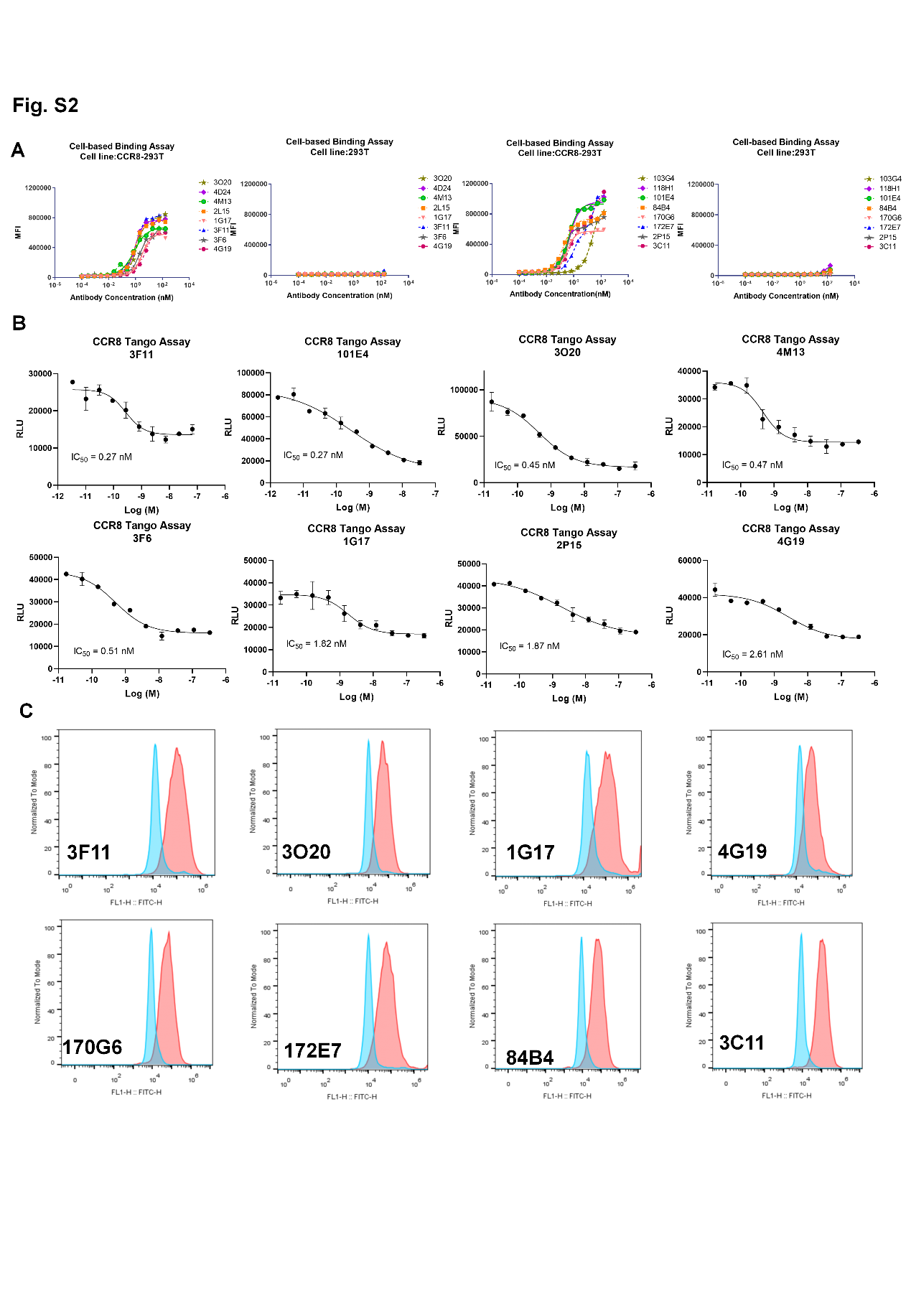
**Fig. S2. Potency and affinity of anti-CCR8 antibodies.**

**(A)** The cell-based binding affinity of different anti-CCR8 antibodies. The binding affinity of these antibodies to CCR8 was measured by FCM. **(B)** Dose-dependent inhibition of CCL1 mediated CCR8 signal activity of different anti-CCR8 antibodies by the tango assay. **(C)** Cross-reactivity with cynomolgus CCR8 of different anti-CCR8 antibodies. Note: 84B4, 170G6, 172E7, 3C11, 3O20, 1G17, 3F11, and 4G19 were able to bind both human and cynomolgus CCR8.


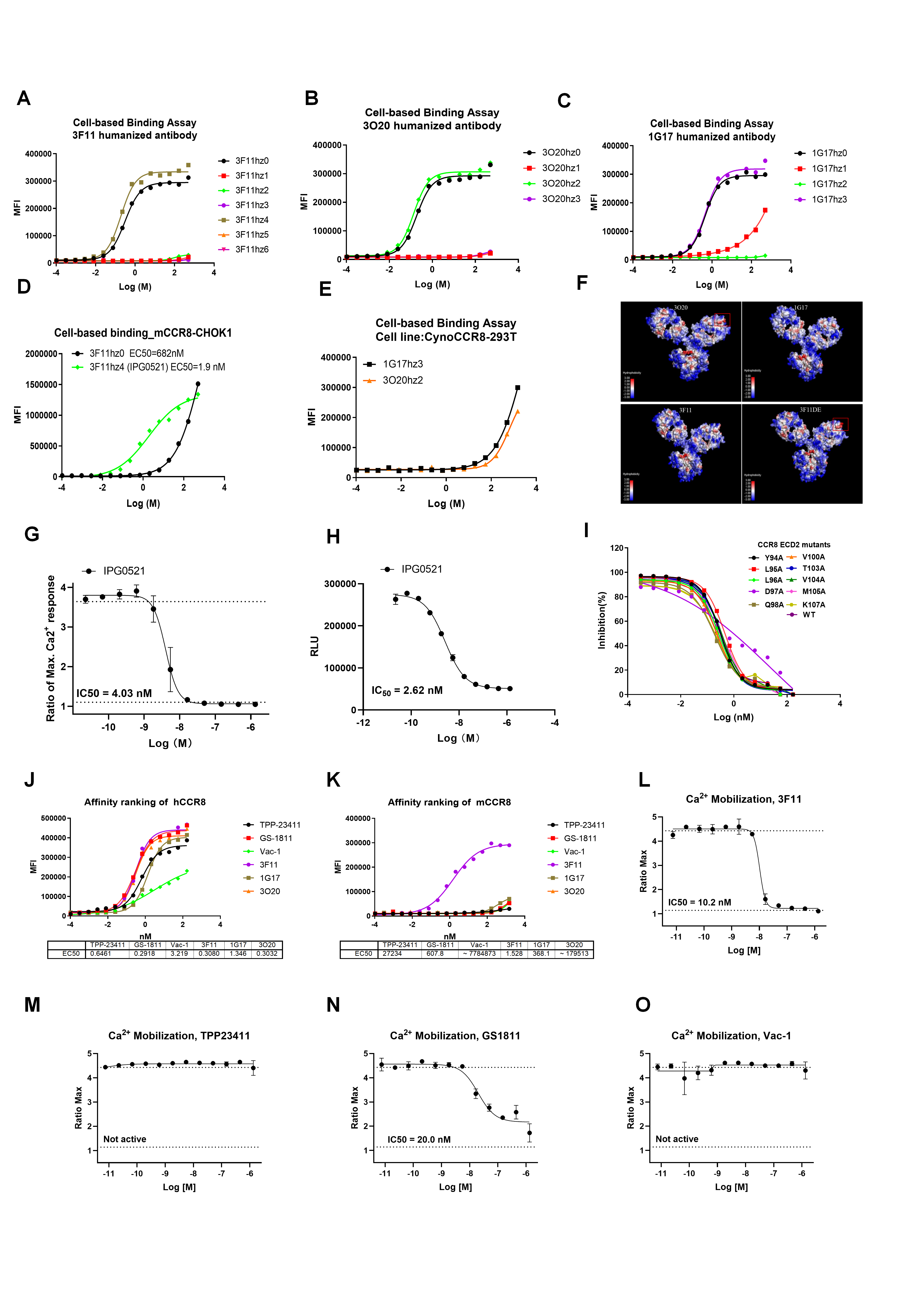


**Fig. S3. *In vitro* profile of IPG0521, compared with** **similar commercial products.**

**(A-C)** The cell-based binding affinity of different anti-CCR8 humanized antibodies. The binding affinity of these antibodies to CCR8 was measured by FCM. The affinity EC50 of 3F11hz4, 3O20hz2, and 1G17hz3 was 0.19, 0.13, and 0.47 nM, respectively. **(D)** The affinity EC50 of 3F11hz4 binding to mouse CCR8 was increased from 681.9 nM to 1.926 nM. **(E)** The affinity EC50 of 3O20hz2 and 1G17hz3 binding to cynomolgus CCR8 were 641 and 3142 nM, respectively. **(F)** Prediction of hydrophobic regions of CCR8 humanized antibodies. ***Note：****3O20hz2 had a hydrophobic region at the Fab, suggesting that the high risk of aggregation and the aggregation peak of 3O20hz2 indeed increased significantly in the later light stability studies.* (**G)** The inhibitory effect of IPG0521 on CCR8-mediated calcium mobilization. IPG0521 effectively inhibited the downstream signaling of CCR8-mediated calcium mobilization induced by CCL1, with an IC50 of 4.03 nM. (**H)** The inhibitory effect of IPG0521 on CCR8-mediated β-arrestin signaling. IPG0521 effectively inhibited the downstream signaling of CCR8-mediated β-arrestin signaling induced by CCL1, with an IC50 of 2.62 nM. (**I)** Epitope analysis of IPG0521. After mutating at 97th amino acid from aspartic acid (D) to alanine (A), the binding affinity EC50 of IPG0521 to CCR8 was dropped from 0.2335 to 23.96 nM. (**J-O)** Affinity test (J-K) and Calcium mobilization assay (L-O) of IPG0521 compared with several related commercial products such as Bayer (TPP-23411), Gilead (GS1811) and Surface (Vac-1).


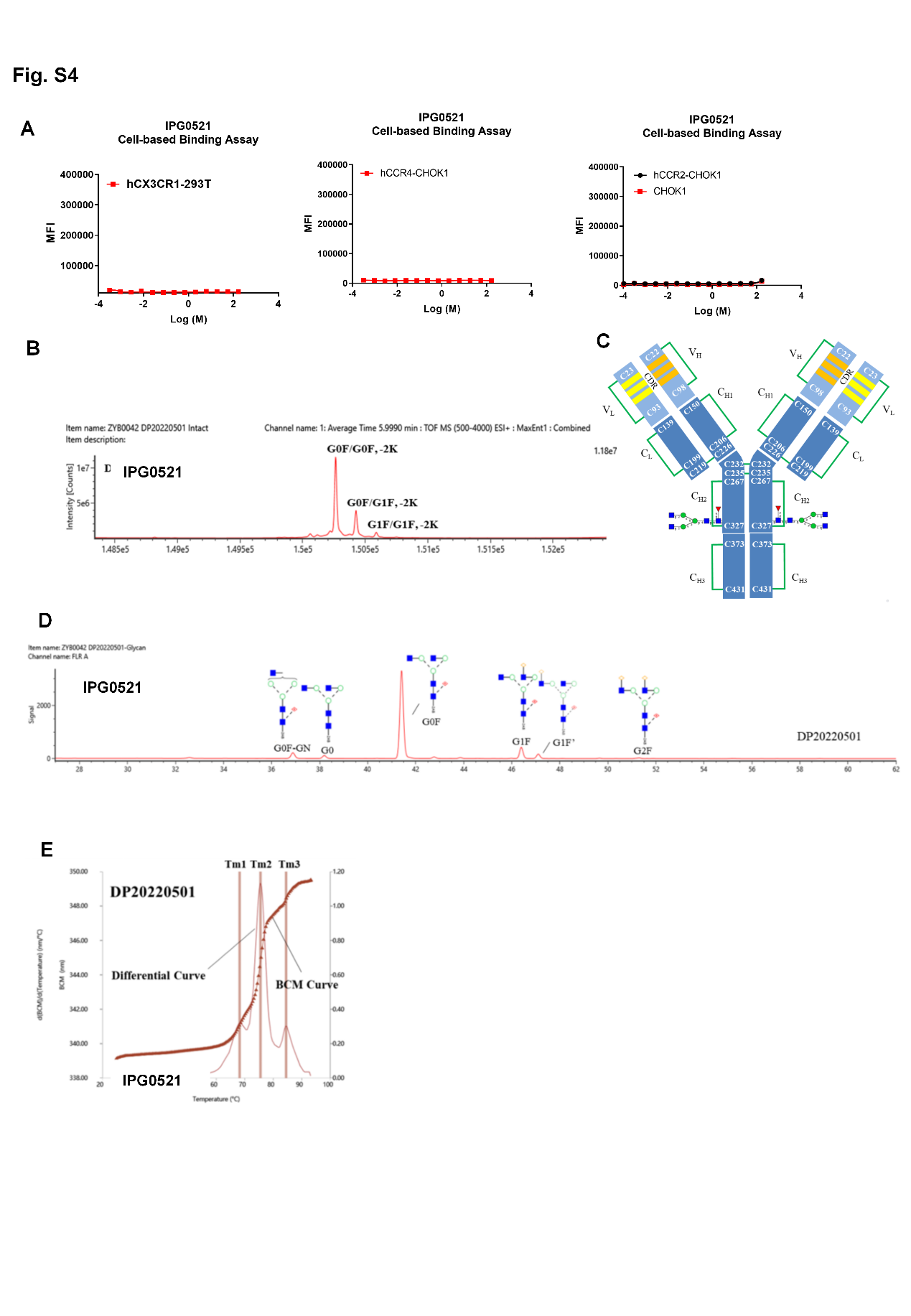
**Fig. S4. The specificity and physicochemical properties of IPG0521.**

**(A)** IPG0521 only specifically binds human CCR8, but not human CCR4, X3CR1, CR2, or 293T cells. **(B)** The mass spectra-intact molecular weight of IPG0521. The intact molecular weight of the IPG0521 samples was determined by quantitative time-of-flight (QTOF) high-resolution mass spectrometry. The samples were diluted to 0.5 mg/mL, desalted, and analyzed on a Xevo G2-XS QTOF mass spectrometer (Waters). **(C)** Schematic of IPG0521. Green lines marked show disulfide bonds; N303 of the heavy chain is the site of *N*-glycosylation in IPG0521. **(D)** The stacked chromatograms of IPG0521. The main glycans identified are G0F and G1F. **(E)** Different scanning fluorimetry traces of IPG0521. The T_m1_ of IPG0521 is 68.7 ℃.


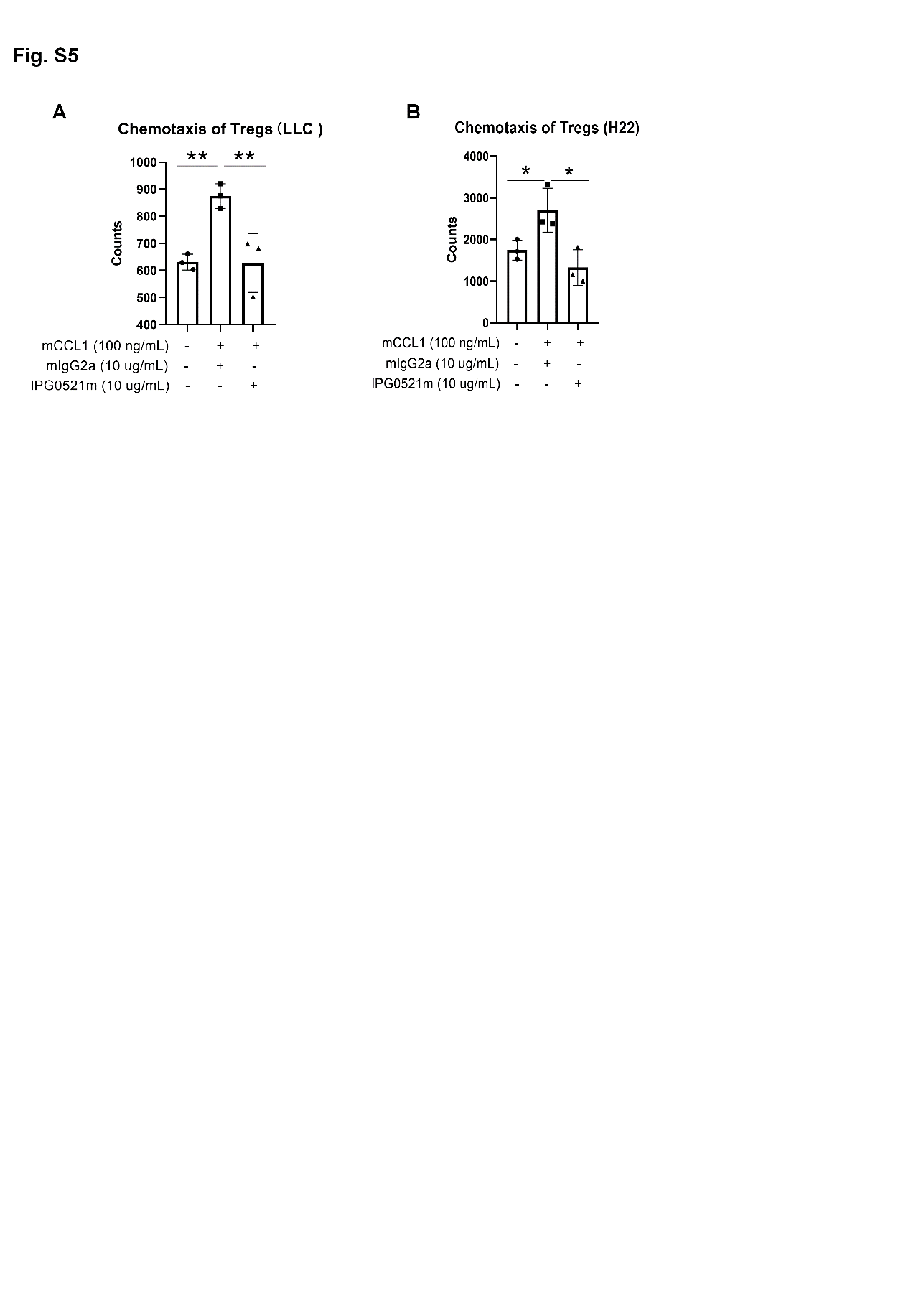
**Fig. S5. IPG0521m blocking CCL1 mediated tumor infiltrated Tregs chemotaxis *in vitro.***

**(A-B)** IPG0521 abolished CCL-1 mediated the migration of Treg cells isolated from the tumor of LLC and H22 syngeneic cancer models by using a trans-well assay. Data were shown as mean ± SD. ******p*＜ 0.05, *******p*＜ 0.01.

**Fig. S6. IHC analysis of immune cells in tumors with or without IPG0521m treatment, and the antitumor effect of IPG0521m in the HepG2 liver cancer model and LLC lung cancer model.**

**(A)** The expression of CD45^+^ immune cells, CD4^+^ cells, CD8^+^ cells, FoxP3^+^ cells, as well as NKP46^+^ cells in tumors with or without IPG0521m treatment was assessed by IHC analysis. **(B)** Quantification of these cells described in (A). **(C)** The tumor growth inhibition rate (TGI) curve and tumor weights and representative image of tumors treated with IPG0521m in an HepG2 mouse model. **(D)** The tumor growth inhibition rate (TGI) curve and tumor weights and representative image of tumors treated with IPG0521m in an LLC mouse model. Data were shown as mean ± SD. ******p*＜ 0.05, *******p*＜ 0.01, ********p*＜ 0.001.

**Fig. S7. IPG0521m has no influence on the proportion of tumor infiltrated DCs.**


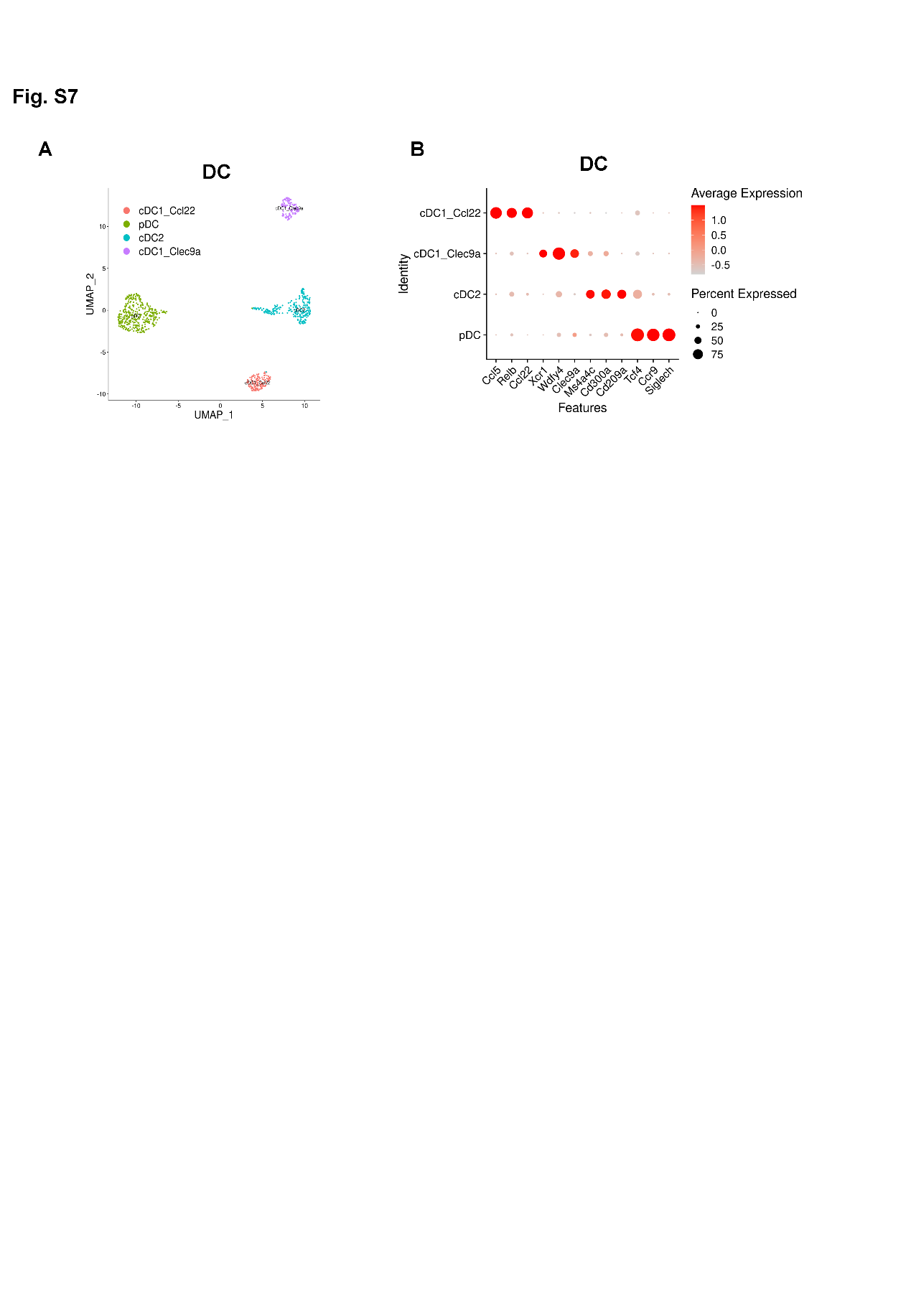
 **(A)** UMAP projection of DC cells with 4 clusters. **(B)** The bubble plot shows the enrichment of distinct markers and DC cell-related signature genes of different DC cell subclusters.

**Tables S1 to S7 for multiple supplementary tables**

Supplementary Table 1. The summary of different immunization Strategies

| Immunization Strategy | Immunogen | Animal No. | Strain | Route | Dosage (/animal) | | Adjuvant | Positive Clones Obtained | Cross with Cynomolgus monkeys | Block CCL1-CCR8 antibodies |
| --- | --- | --- | --- | --- | --- | --- | --- | --- | --- | --- |
| I | hCCR8-CHOK1 | 8736, 8737,8738, 8739, 8740 | SJL | i.p.  with immune tolerance breaking | 0.5-1x10^7^ | N/A | | 0 | 0 | 0 |
| II | CCR8 DNA | 8691, 8692, 8693, 8694, 8695 | SJL | Gene gun | 4 ug | GM-CSF & FLT3L | | 6 | 3 | 3 |
|  | hCCR8-293T |  |  |  |  |  |  |  |  |  |
|  |  |  |  | i.p. | 0.5-1x10^7^ | N/A | |  |  |  |
| III | hCCR8-293T | 03-M2, 04-M1, M2, 05-M3, 06-M3, 377, 380 | SJL | i.p. and pedicle injection | 2 x 10^7^ | N/A | | 10 | 5 | 10 |
| IV | hCCR8-293T membtane protein | CpG-M1, M2, Sigma-M1, M2 | SJL | Intramuscular injection or pedicle injection | 100 ug | CpG+Al & Sigma adjuvant | | 0 | 0 | 0 |

Supplementary Table 2: EC50s of the antibodies

| **Clone** | **Binding Affinity of hCCR8 EC50 (nM)** |
| --- | --- |
| 103G4 | 20.56 |
| 118H1 | 0.4664 |
| 101E4 | 0.4088 |
| 84B4 | 0.195 |
| 172E7 | 6.989 |
| 2P15 | 0.3052 |
| 3C11 | 4.254 |
| 170G6 | 0.3021 |
| 3O20 | 1.293 |
| 4M13 | 0.775 |
| 2L15 | 0.8257 |
| 4D24 | 0.888 |
| 1G17 | 2.939 |
| 3F11 | 0.9572 |
| 3F6 | 2.293 |
| 4G19 | 4.487 |

Supplementary Table 3: Stability of 3O20, 1G17, and 3F11 humanized antibodies

| Stress Condition | Humanized Ab | SEC | | |
| --- | --- | --- | --- | --- |
|  |  | Aggregate% | Main peak% | Fragments% |
| 0 | 3O20-F01 | 3.5 | 96.5 | N/A |
|  | 1G17-F02 | 0.2 | 99.8 | N/A |
|  | 3F11-F03 | 0.2 | 99.8 | N/A |
| PhoD3 | 3O20-F01 | 7.0 | 92.9 | 0.1 |
|  | 1G17-F02 | 1.3 | 98.6 | 0.1 |
|  | 3F11-F03 | 1.2 | 98.6 | 0.2 |
| PhoD7 | 3O20-F01 | 9.3 | 89.5 | 1.2 |
|  | 1G17-F02 | 3.3 | 94.9 | 1.8 |
|  | 3F11-F03 | 2.6 | 95.0 | 2.3 |
| 40°C D7 | 3O20-F01 | 4.1 | 93.7 | 2.3 |
|  | 1G17-F02 | 0.6 | 96.8 | 2.6 |
|  | 3F11-F03 | 0.4 | 97 | 2.7 |
| 40°C D14 | 3O20-F01 | 3.9 | 94.1 | 2.1 |
|  | 1G17-F02 | 0.7 | 97.2 | 2.1 |
|  | 3F11-F03 | 0.5 | 97.2 | 2.3 |
| 40°C D28 | 3O20-F01 | 4 | 93.7 | 2.7 |
|  | 1G17-F02 | 1 | 97 | 2 |
|  | 3F11-F03 | 0.6 | 97.3 | 2.1 |

Supplementary Table 4: CCR8 gene homology across different species

| **CCR8** | **Human**  ***vs.*rat** | **Human**  ***vs.*dog** | **Human**  ***vs.* mouse** | **Human**  ***vs.*cynomolgus** |
| --- | --- | --- | --- | --- |
| ECD1 | 59% | 71% | 60% | 77% |
| ECD2 | 100% | 93% | 92% | 93% |
| ECD3 | 59% | 84% | 65% | 97% |
| ECD4 | 60% | 67% | 60% | 94% |

Supplementary Table 5: Binding activity of IPG0521 in mutant CCR8

| Mutant | Inhibition EC50 (nM) |
| --- | --- |
| hCCR8(Y94A) | 0.3064 |
| hCCR8(L95A) | 0.4588 |
| hCCR8(L96A) | 0.2796 |
| hCCR8(D97A) | 23.96 |
| hCCR8(Q98A) | 0.194 |
| hCCR8(V100A) | 0.2368 |
| hCCR8(T103A) | 0.3114 |
| hCCR8(V104A) | 0.3414 |
| hCCR8(M105A) | 0.3338 |
| hCCR8(K107A) | 0.22 |
| hCCR8 | 0.2335 |

Supplementary Table 6: Primer sequences for quantitative real-time PCR

| Gene | Forward primer sequence (5’-3’) | Reverse primer sequence (5’-3’) |
| --- | --- | --- |
| mGAPDH | AGGTCGGTGTGAACGGATTTG | TGTAGACCATGTAGTTGAGGTCA |
| mLag3 | CCTCGATGATTGCTAGTCCCT | GTAGACAGGCACTCGGTTCTG |
| mCtla4 | AGTGGGCTTCCTAGATTACCC | GTCCCGTGTCAACAGCTCTC |

m: mouse

Supplementary Table 7: Antibodies used in flow cytometry.

| Antibodies | Vendor | Catalog | Lot |
| --- | --- | --- | --- |
| FITC anti-mouse CD45 | Biolegend | 103108 | B363912 |
| PC7 anti-mouse CD3 | Biolegend | 100220 | B356288 |
| BB700 anti-mouse CD4 | BD Biosciences | 566407 | 2096354 |
| BV421 anti-mouse Foxp3 | BD Biosciences | 562996 | 2033214 |
| PE anti-mouse CD198 (CCR8) | Biolegend | 150312 | B377338 |
| BB700 anti-mouse CD8α | BD Biosciences | 566409 | 2172032 |
| BV421 anti-mouse IFN-γ | Biolegend | 505830 | B372834 |
| PB450anti-mouse CD279 (PD-1) | Biolegend | 135218 | B349792 |
| PE anti-mouse CD366 (Tim-3) | Biolegend | 134004 | B294038 |
| PE anti-mouse CD335 (NKp46) | Biolegend | 137604 | B261049 |
| APCanti-mousePerforin | Biolegend | 154304 | B366020 |

**Supplementary materials and methods**

1. **Production of mouse monoclonal antibodies by the hybridoma technology**
2. **Construction of CCR8 overexpression cell lines**

The coding sequences of human CCR8, rat CCR8, dog CCR8, and cynomolgus CCR8 were cloned into pLVX-vector, generating the lentivirus particles. 293T cells were transfected respectively with human, rat, dog, and cynomolgus CCR8 lentivirus particles by polybrene (8 μg/ml), selected in media containing puromycin (2 μg/ml, P8230, solarbio). CHO-K1 cells were transfected respectively with human and mouse CCR8 lentivirus particles by polybrene (8 μg/ml), selected in media containing puromycin (6 μg/ml). All overexpression cell lines were tested for the expression of CCR8 by FCM. Individual clones with the greatest MFI to CCR8 were selected for subsequent studies.

1. **Animal Immunization**

All procedures involving the care and use of animals in the study were reviewed and approved by the Immunophage Institutional Animal Care and Use Committee (IACUC protocol No.: IMP-SH220-A1). During the study, the care and use of animals were conducted in accordance with the guides of the Association for Assessment and Accreditation of Laboratory Animal Care International (AAALAC). Three different immunization protocols were used in this study. The cellular immunization protocol was tried with different combinations of cell concentration and injection route. The human CCR8-293T cells or hCCR8-CHOK1 were resuspended in DPBS at 2x10^7^/ml and used to immunize SJL mice of about 6-8 weeks old via the abdominal and pedicle injections every two weeks. During the fast immunization procedure, these animals were immunized about 3-4 times. The second method used a gene gun for immunization with CCR8 DNA in GM-CSF&FLT3L adjuvant, 4 μg each time, every 2 weeks for 3 consecutive immunizations followed by cellular immunization, 0.5-1x10^7^ cells volume per intraperitoneal injection, every 2 weeks for 4 consecutive injections. The third method was immunization with membrane proteins extracted from hCCR8-293T. Two different water-soluble adjuvants, CpG-B DNA (HC4039-200 nmol, Hycult) or Sigma adjuvant system (S6322-1VL, sigma) were mixed with 100 μg of protein each time, intramuscular or pedal injection once a week.

1. **Serum titer measurement**

After immunization, animals were selected by tittering using FCM. Briefly, the serum from immunized mice was diluted from 1:100 to 1:1800. Approximately 5×10^5^ CCR8-CHO-K1 cells and CHO-K1 cells per sample were prepared and blocked with mouse TruStain FcX™ PLUS (156604, Biolegend). The cells were distributed into 96-well round-bottom polystyrene plates and incubated with the diluted serum for 20-30 minutes. Next, the cells were washed with PBS/0.5% BSA and centrifuged. The pelleted cell samples were then incubated with the second antibody of anti-mouse IgG labeled with FITC at 1:300 dilution in 100 μl PBS/0.5% BSA on ice for 30 minutes, and then washed with PBS/0.5% BSA and spun down. The cell pellets were resuspended in PBS/0.5% BSA, and the samples were analyzed on a CYTOFLEX (Beckman). The titer of each mouse was determined by the MFI ratio (MFI_CCR8-CHOK1_/MFI_CHOK1_). Immunized mice with the highest titer were sacrificed. Spleen and lymph node cells were suspended in DMEM before fusion with a myeloma cell line Sp2/0-Ag14.

1. **Cell fusion**

Spleen and lymph node cells were fused with Sp2/0-Ag14 by PEG (P7181, sigma). The fused cells were suspended in HAT selecting medium (21060017, Gibco) and distributed into 384-well plates. The medium was replenished on day 4 or 8 with a fresh one.

1. **Hybridoma high throughput screening and sub-cloning**

After 10 days of culturing, hybridoma supernatants were screened for CCR8-specific monoclonal antibodies. Supernatants containing antibodies were tested by cell-based ELISA for reactivity with CCR8 overexpressing cells. Supernatants of these positive clones were then confirmed by FCM. Generally, the same supernatants were tested on non-transfected parental cells to confirm that the reactive antibody recognized CCR8 specifically. After the selection of positive cell pools, sub-cloning was done by limiting dilution.

1. **Monoclonal antibody isotype identification and hybridoma sequencing**

SBA clonotyping system-HRP Kit (5300-05, SouthernBiotech) was used to identify the isotype of the monoclonal antibodies. Hybridoma sequencing was performed by GENWIZ.

**2 Tango Assay**

Tango assays were carried out to determine which of the antibodies could block the CCL1-CCR8 signal. Tango-CCR8-Gal4-CHO-K1 cells were pelleted and resuspended at 1×10^4^ cells/70 μl/well. The cells were distributed into 96 well plates, incubated in starving medium (F12K, 1% FBS, 1% penicillin-streptomycin) in 5% CO_2_ at 37℃ for 6 hours, the test antibodies were added and incubated for 1 hour, and then CCL1 (272-I, R&D) was added and incubated in 5% CO_2_ at 37℃ for 24 hours. After overnight culture, the cells were incubated with ONE-Glo working reagent at room temperature in the dark for 10 minutes, and then the relative luminescence units (RLU) of each sample were measured using a microplate luminescence reader at 560 nm and recorded.

**3 Calcium Mobilization Assay**

The hCCR8-Gqi5-293T cells passed in a complete medium (DMEM, 10% FBS, 1% penicillin-streptomycin, 0.75 μg/ml puromycin, 400 μg/ml G418) in an incubator (37℃, 5% CO_2_) were used in the Calcium mobilization assay. The fluorescent membrane-permeable calcium-binding dye (R8190, Molecular Devices) was dissolved in assay buffer (20 mM HEPES buffer with HBSS, pH 7.4). The loading buffer was prepared with the dye solution containing 5 mM probenecid (HY-B0545, MCE). The probenecid was prepared into 500 mM stock solution in 1 N NaOH and then diluted to 250 mM in HBSS buffer before use.

Approximately 1.5x10^4^ hCCR8-Gqi5-293T cells were seeded into a 384-well plate and incubated in 25 μl starving medium (DMEM, 1% FBS, 1% penicillin-streptomycin) in 5% CO_2_ at 37℃ for 16 hours. Then, the starving medium was completely changed with 25 μl assay buffer, and 25 μl loading buffer was added to the desired wells. After adding dye, the cell plate was incubated for 2 hours at 37°C with 5% CO_2_ and then kept at room temperature until used. The humanized antibodies in 12.5 μl assay buffer at the desired concentration (5×) were added into each well and incubated with cells for 30 minutes at room temperature. After incubation, the microplate was transferred to the FLIPR instrument, and the calcium assay was started as described in the user guide for the instrument. 12.5 μl assay buffer with or without CCL1 was added during the assay. The MAX ratio value was plotted against the antibody concentration and analyzed in GraphPad Prism for concentration curve generation.

**4 ADCC assays**

The hCCR8-Gqi5-293T cells passed in a complete medium (DMEM, 10% FBS, 1% penicillin-streptomycin, 0.75 μg/ml puromycin, 400 μg/ml G418) in an incubator (37℃, 5% CO_2_) were used in the ADCC assay. The ADCC FcγRIIIa (158V) Jurkat effector cells passed in a complete medium (RPMI 1640, 10%FBS, 1% penicillin-streptomycin, 0.75 μg/ml puromycin, 3.5 μg/ml blasticidin) in an incubator (37℃, 5% CO_2_).

Approximately 1x10^4^ hCCR8-Gqi5-293T cells were seeded into each well of a black/clear 96-well plate with 100 μL complete medium and incubated at 37℃, 5% CO_2_ overnight. The tested antibodies were diluted in gradient with a 1:4 dilution ratio using ADCC reaction buffer. The ADCC FcγRIIIa (158V) Jurkat effector cells were washed once with ADCC reaction buffer and were resuspended in ADCC reaction buffer at the concentration of 3×10^6^ cells/ml. The culture medium of target cells was removed, then 50 μL of ADCC effector cells (3×10^6^ cells/ml) and 50 μL of serial dilution of IPG0521 were added into each well. Cell plates were incubated at 37℃, 5% CO_2_ for 6 h. One-Glo detection buffer (E6120, Promega) was prepared according to instructions of the manufacturer, and 100 μL of prewarmed One-Glo detection buffer was added into each well. After 10 min of incubation at room temperature, the luminescence (RLU) was measured with a microplate reader. The results were analyzed using GraphPad Prism software.

1. **Humanization of antibodies**

Antibodies were humanized by grafting the CDRs of lead antibodies into selected human IgG germline frameworks. The human germline was selected based on sequence similarity within both frameworks (FR). To maintain canonical loop structure and chain interface, certain residues in human germline frameworks were back mutated to corresponding mouse residues. The humanized antibodies' heavy and light chains were synthesized and inserted into the pcDNA3.4 vector to construct expression plasmids of full-length IgGs. Expression of antibodies was conducted in CHO-K1 cell culture. The humanized antibodies were confirmed to bind to human CCR8-293T, mouse CCR8-CHO-K1, rat CCR8-293T, dog CCR8-293T, and cynomolgus CCR8-293T by FCM.

1. **Stability of humanized antibody**

Stability studies of humanized antibodies include high-temperature stability studies (40±2°C/75±5% RH) and light stability studies (total illumination of light source not less than 1.2×10^6^ Lux-hr, near UV lamp energy not less than 200 W-hr/m^2^). For the high-temperature stability study, samples were collected after being placed at 40 °C for 7, 14, and 28 days. For light stability studies, samples were collected after being placed under light conditions for 3 days and 7 days. The samples were then diluted to 10 mg/mL with mobile phase, centrifuged at 12, 000 rpm for 5 min (2-8°C), and the supernatant was taken into a liquid phase vial and injected into the sample using high-performance liquid chromatography, and the chromatograms were recorded to analyze the aggregation, main peaks, and fragment sizes of the samples.

1. **Structure and other characteristics of humanized antibody**
2. **The intact molecule**

The intact molecular weight of the sample was determined by quantitative time-of-flight (QTOF) high-resolution mass spectrometry. The sample was diluted to 0.5 mg/mL, desalted, and analyzed on a Xevo G2-XS QTOF mass spectrometer (Waters). The data were deconvoluted using UNIFI software (Waters).

1. **Disulfide bond**

Samples were denatured by guanidine hydrochloride, blocked by NEM (N-ethyl maleimide), and then digested by Trypsin and Lys-C under non-reducing conditions. The samples were analyzed by Q-TOF RP-LC-MS. Raw data were processed using UNIFI software (Waters). The numbers and positions of disulfide bonds were determined by comparing the theoretical and experimental molecular weights of the enzymatically digested peptides.

1. **N-Glycan analysis**

Glycosylation of therapeutic proteins plays a critical role in *in vivo* activity, contributes to serum half-life, and can affect the immunogenicity of a product. Therefore, the N-glycan analysis provides information on the glycan profile of a protein. Samples were digested with PNGase F to release the N-linked oligosaccharides. The proteins were precipitated and removed from the sample by centrifugation. The supernatant was taken, dried, and labelled with Aminobenzamide (2-AB). Labelled oligosaccharides were separated by HILIC chromatography using an ACQUITY UPLC Glycan BEH Amide column (Waters). Oligosaccharides were detected by FLD detectors and mass spectrometry detectors (Xevo G2 XS, Waters). Each individual N-glycan was quantified by its relative peak area.

1. **Thermal stability**

A differential scanning fluorimeter (DSF) was used to detect the denaturation temperature T_m_ of sample proteins. By increasing the sample temperature linearly, the protein is denatured, and the hydrophobic area is exposed. The conformation change can be monitored by measuring the intrinsic fluorescence (IF) from hydrophobic residues tryptophan and tyrosine. The changes in the fluorescence signal of the protein during the heating process enable the identification of Tm, which indicates thermal stability and thermodynamic characteristics of the protein in solution.

1. **Epitope analysis of humanized monoclonal antibody**

Epitope validation was performed for humanized monoclonal antibodies. The binding evaluation was performed by transiently expressing each mutant of ECD2 in 293T cells and reacting the mutant with an antibody solution of a humanized antibody prepared by 14 serially diluting by 3-fold from 25 μg/ml. After reacting at 4℃ for 1 hour, it was reacted with Alexa Fluor 488 affinipure goat anti-human Ig(H+L) (109-545-003, Jackson), and flow cytometry analysis was performed. The highest MFI of serial dilution is supposed to be 100%. The inhibition percent was calculated according to the following formula.

Inhibition %= (Highest MFI-MFI)/Highest MFI *100%

1. **Immunofluorescence**

The fixed tissues were incubated in 30% sucrose in PBS overnight. The tissues were embedded in the optimal cutting temperature compound (Tissue-Tek; Sakura) and stored at −80 ℃. Slices of 5μm thickness were prepared by using a Leica CM1950 cryotome. Permeabilized by incubating them in 0.5% Triton in PBS for 10 min at room temperature, the slides were blocked in 10% Serum of goat for 1 h at room temperature, incubated with the corresponding primary antibodies overnight at 4 ℃ in a humidified chamber, and then incubated with the secondary antibodies for 1 h at room temperature. Nuclei were stained with Hochest33342 (C1022, Beyotime) for 10 min at room temperature. The slides were then washed, and coverslips were mounted using ProLong Gold anti-fade reagent (Sangon, Shanghai). Fluorescent images were obtained using a Leica microscope (Leica, Wetzlar, Germany). The relevant parameters were determined in five–eight areas in each tumor. Image analysis was performed by thresholding for positive staining and normalizing the tissue area using ImageJ software. Primary antibodies used included anti-CCR8 (ab140796, Abcam) and anti-Foxp3 (ab36607, Abcam); the secondary antibodies used were Alexa-fluor-594 donkey anti-rabbit (1890862, Invitrogen) and Alexa-fluor-488 goat anti-mouse (1964382, Invitrogen).

1. **Bulk RNA sequencing**

RNA quantification and qualification： At Novogene, total RNA was extracted, and its integrity was assessed using the RNA Nano 6000 Assay Kit of the Bioanalyzer 2100 system (Agilent Technologies, CA, USA，5067-1511) following the manufacturer's instructions.

Library preparation for Transcriptome sequencing： The mRNA library was constructed by Novogene following their standard operating procedures (SOP). Briefly, mRNA was purified from total RNA using poly-T oligo-attached magnetic beads. Fragmentation was carried out using divalent cations under elevated temperature in the First Strand Synthesis Reaction Buffer(5X). The first strand cDNA was synthesized using a random hexamer primer and M-MuLV Reverse Transcriptase (RNase H-). The second strand cDNA synthesis was subsequently performed using DNA Polymerase I and RNase H. Remaining overhangs were converted into blunt ends via exonuclease/polymerase activities. After the adenylation of 3' ends of DNA fragments, an Adaptor with a hairpin loop structure was ligated to prepare for hybridization. The library fragments were purified with the AMPure XP system (Beckman Coulter, Beverly, USA) to select cDNA fragments of preferentially 370~420 bp in length. Then, PCR was performed with Phusion High-Fidelity DNA polymerase, Universal PCR primers, and Index (X) Primer. At last, PCR products were purified (AMPure XP system), and library quality was assessed on the Agilent Bioanalyzer 2100 system.

Clustering and sequencing (Novogene Experimental Department): The clustering of the index-coded samples was performed on a cBot Cluster Generation System using TruSeq PE Cluster Kit v3-cBot-HS (Illumia) according to the manufacturer's instructions. After cluster generation, the library preparations were sequenced on an Illumina Novaseq platform, and 150 bp paired-end reads were generated.

Quality control： Raw data (raw reads) in fastq format were first processed through in-house Perl scripts. In this step, clean data (clean reads) were obtained by removing reads containing adapter, reads containing ploy-N, and low-quality reads from raw data. At the same time, Q20, Q30, and GC content of the clean data were calculated. All the downstream analyses were based on clean data with high quality.

Reads mapping to the reference genome： Reference genome and gene model annotation files were downloaded directly from the genome website. The index of the reference genome was built using Hisat2 v2.0.5, and paired-end clean reads were aligned to the reference genome using Hisat2 v2.0.5.

FeatureCounts v1.5.0-p3 was used to count the reads numbers mapped to each gene. Then, the FPKM of each gene was calculated based on the length of the gene and the read count mapped to this gene. Differential expression analysis of two conditions/groups (two biological replicates per condition) was performed using the DESeq2R package (1.20.0). Gene Ontology (GO) enrichment analysis of differentially expressed genes was implemented by the clusterProfiler R package, in which gene length bias was corrected. GO terms with corrected P value less than 0.05 were considered significantly enriched by differentially expressed genes. We used the local version of the GSEA analysis tool http://www.broadinstitute.org/gsea/index.jsp, GO, and KEGG data sets were used for Gene Set Enrichment Analysis (GSEA) independently.

1. **Single-cell RNA Statistical Analysis**

scRNA-seq data analysis was performed by NovelBio Co., Ltd. with NovelBrain Cloud Analysis Platform (www.novelbrain.com). We applied fastp with default parameter filtering the adaptor sequence and removed the low-quality reads to achieve clean data. Then, the feature-barcode matrices were obtained by aligning reads to the mouse genome (mm10 Ensemble: version 100 using CellRanger v6.1.1. We applied the down sample analysis among samples sequenced according to the mapped barcoded reads per cell of each sample and finally achieved the aggregated matrix. Cells containing over 200 expressed genes and mitochondria UMI rate below 20% passed the cell quality filtering, and mitochondria genes were removed in the expression table.

Seurat package (version: 4.0.3, https://satijalab.org/seurat/) was used for cell normalization and regression based on the expression table according to the UMI counts of each sample and percent of mitochondria rate to obtain the scaled data. PCA was constructed based on the scaled data with the top 2000 high variable genes, and the top 10 principals were used for tSNE construction and UMAP construction. Using the graph-based cluster method, we acquired the unsupervised cell cluster result based on the PCA top 10 principal and calculated the marker genes by the FindAllMarkers function with the Wilcox rank sum test algorithm under the following criteria:1. log2FC > 0.25; 2. pvalue<0.05; 3. min.pct>0.1. In order to identify the cell type in detail, the clusters of the same cell type were selected for re-tSNE analysis, graph-based clustering, and marker analysis.

**QuSAGE Analysis (Gene Enrichment Analysis)**

To characterize the relative activation of a given gene set, such as pathway activation, "Antigen processing and presentation," and "Chemokine signaling pathway," as described before, we performed QuSAGE (2.16.1) analysis.

**Differential Gene Expression Analysis**

To identify differentially expressed genes among samples, the function FindMarkers with Wilcox rank sum test algorithm was used under the following criteria:1. lnFC > 0.25; 2. pvalue<0.05; 3. min.pct>0.1

**Go Analysis**

Gene ontology (GO) analysis was performed to facilitate elucidating the biological implications of marker genes and differentially expressed genes. We downloaded the GO annotations from NCBI (http://www.ncbi.nlm.nih.gov/), UniProt (http://www.uniprot.org/) and the Gene Ontology (http://www.geneontology.org/). Fisher's exact test was applied to identify the significant GO categories, and FDR was used to correct the p-values.

**Pathway Analysis**

Pathway analysis was used to determine the significant pathway of the marker genes and differentially expressed genes according to the KEGG database. We turned to Fisher's exact test to select the significant pathway, and the threshold of significance was defined by P-value and FDR.

1. **Mouse in vivo experiment**
2. **LLC tumor model**

LLC cells were maintained in DMEM high glucose medium supplemented with 10%FBS at 37℃ under 5% CO_2_. Lewis lung carcinoma (LLC) cells (1 x 10^6^/mouse) were inoculated subcutaneously at the right flank abdomen of female C57BL/6 mice to establish the transplanted tumor model. When the average volume of the transplanted tumor reached about 50-100 mm^3^, 40 tumor-bearing mice with similar tumor volume were randomly grouped and intraperitoneally injected with isotype control or different doses of IPG0521m (1, 3, 10 mg/kg) twice a week.

1. **H22 tumor model**

H22 cells were maintained in DMEM medium supplemented with 10%FBS at 37℃ under 5% CO_2_. H22 cells (1 x 10^6^/mouse) were inoculated subcutaneously at the right flank abdomen of female Balb/c mice to establish the transplanted tumor model. When the average volume of the transplanted tumor reached about 50-100 mm^3^, 20 tumor-bearing mice with similar tumor volume were randomly grouped and intraperitoneally injected with isotype control or IPG0521m (10 mg/kg) twice a week. Tumor volume and body weight were measured twice a week.

1. **IPG0521m withdrawal in H22 tumor model**

For testing the prolonged antitumor effect of IPG0521m, 3 groups of mice were designed in a similar way as before. Twenty-four tumor-bearing mice with similar tumor volume were randomly grouped and intraperitoneally injected with isotype (mIgG2a), IPG052m withdrawal (10 mg/kg), and IPG0521m (10 mg/kg) twice a week. On Day 22, the IPG0521m withdrawal group (10 mg/kg) stopped the administration of drugs. Tumor volume and body weight were measured twice a week.

1. **IPG0521m in combination with αPD-1 in H22 tumor model**

The cell culture and inoculation process for H22 is the same as before. Forty tumor-bearing mice with similar tumor volumes were randomly grouped and intraperitoneally injected with isotype (mIgG2a), IPG0521m (10 mg/kg), αPD-1(3 mg/kg), and IPG0521m (10 mg/kg) combined with αPD-1(3 mg/kg) twice a week.

At the end of the experiment, the efficacy was evaluated by calculating the tumor growth inhibition rate (TGI); the tolerability was evaluated by observing the weight loss, death, and treatment-related clinical symptoms. One-way ANOVA and Tukey's multiple comparisons were performed to compare the statistical difference between the drug-administered group and the vehicle group. The amendments were approved by the IACUC Committee (No.: IMP-SH19-T1).
